# Oral Engineered Extracellular Vesicles Based on Ion Exchange Strategy for Multipronged Management of Wilson's Disease Complicated with Reproductive Dysfunction Therapy

**DOI:** 10.1002/advs.202501689

**Published:** 2025-07-17

**Authors:** Tingting Wang, Wengui Lu, Zhifei Cheng, Luyao Wang, Zhenzhen Jiang, Yike Yue, Pengyu Jiang, Zehua Xia, Lei He, Fengying Wang, Limin Wu, Qi Wang, Hui Han

**Affiliations:** ^1^ Department of Neurology The First Affiliated Hospital of Anhui University of Chinese Medicine Hefei Anhui 230031 China; ^2^ Anhui Province Key Laboratory of Pharmaceutical Preparation Technology and Application School of Pharmacy Anhui University of Chinese Medicine Hefei Anhui 230012 China; ^3^ Center for Reproduction and Genetics The First Affiliated Hospital of USTC Division of Life Sciences and Medicine University of Science and Technology of China Hefei Anhui 230001 China

**Keywords:** external copper inhibition, intracellular copper chelation, ion exchange strategy, neuroinflammation, oral drug delivery system, wilson's disease

## Abstract

Wilson's disease (WD), as a typical disease of excessive Cu^2+^ deposition, is characterized by disorders of copper metabolism in the brain and thereby damaging the reproductive system. Conventional interventions using copper chelators can temporarily reduce intracellular copper (in‐copper), but continuous re‐entry of extracellular copper (ex‐copper) into cells results in suboptimal therapy. Effective therapy requires multipronged copper metabolism management. Here, an orally administered, engineered Ganoderma‐derived extracellular vesicle (CZGE) is developed for synergistic in/ex‐copper regulation. Specifically, CZGE is designed by co‐fusing Ganoderma‐derived extracellular vesicle (GEVs) and targeted nanomicelles containing zinc‐curcumin (Zn‐Cur) complex (ZCNs). CZGE, with *β*‐glucan‐enriched GEVs and kisspeptin‐10‐modified ZCNs, can effectively penetrate intestinal and brain barriers after oral administration to target hypothalamic neurons.After internalization, Zn‐Cur released from CZGE replaces excess Cu^2+^ to form copper‐curcumin (Cu‐Cur) and release Zn^2+^ via ion exchange. Cu‐Cur reduces in‐copper and neuroinflammation via the Nrf2/NLRP3 pathway, while Zn^2+^ inhibits ex‐copper influx by activating zinc transporter 1. In WD mice, CZGE alleviates hypothalamic copper deposition, activates ERK1/2 phosphorylation, and repairs reproductive dysfunction by modulating the hypothalamic‐pituitary‐testicular axis. It first reveals a targeted nanomedicine based on ion exchange that multipronged management in‐copper and ex‐copper, offering a promising therapeutic strategy for addressing WD with reproductive dysfunction.

## Graphical abstract

This study reports for the first time an oral targeted nanomedicine that conducts ion exchange through engineered Ganoderma‐derived extracellular vesicle, managing both the in‐copper and ex‐copper in a multi‐pronged approach, offering a promising therapeutic strategy for addressing WD with reproductive dysfunction.

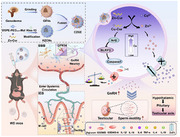

## 1 Introduction

Wilson's disease (WD) is a severe dysregulation of copper metabolism in the brain caused by mutations in the ATP7B gene, characterized by the abnormal accumulation of copper ions (Cu^2+^) and thereby impairing the neuron biofunction.^[^ ^1^ ^]^ Among them, gonadotropin‐releasing hormone (GnRH) neuron, a key of neuron in the hypothalamus that controls reproduction by hypothalamic‐pituitary‐gonadal (HPG) axis, mediates the secretion of GnRH through the intracellular key protein extracellular regulated protein kinases 1/2 (ERK1/2).^[^ ^2^ ^]^ Once GnRH neuron apoptosis/death mediated by Cu^2+^ overload in the hypothalamus of WD, the secretion of GnRH can be put on brake, leading to the inevitable cascade of reproductive dysfunction.^[^ ^3^ ^]^ Therefore, effective removal of excess Cu^2+^ in damaged GnRH neurons is currently the most routine strategy in preclinical and clinical application.^[^ ^4^ ^]^ However, there is a major limitation in the current Cu^2+^ chelation strategy that poor intracellular chelation, rapid clearance, and high systemic toxicity of small molecule chelators, leading to inadequate intracellular chelation effects.^[^ ^5^ ^]^ Besides, existing therapies primarily focus on eliminating excess intracellular copper (in‐copper), while overlooking the continuous accumulation of extracellular copper (ex‐copper), which serves as a significant source of in‐copper.^[^ ^6^ ^]^ It poses a substantial challenge to achieving long‐term therapeutic efficacy. These limitations put forward higher requirements for the design of copper regulation strategy for WD with reproductive dysfunction therapy.

Targeted ion‐regulation therapy based on nanotechnology enables precise and efficient reversal of the ion distribution status in target cells, thereby circumventing the inherent physicochemical properties of pure chelators.^[^ ^7^ ^]^ The ion exchange strategy, among various ion‐regulation approaches, facilitates the separation and replacement of distinct ions by leveraging the affinity and selectivity of suitable ion exchangers for different ions.^[^ ^8^ ^]^ It exhibits great clinical potential in treating WD complicated with reproductive dysfunction as it not only eliminates harmful in‐copper but also introduces other functional ions.^[^ ^9^, ^10^ ^]^ Zinc ion (Zn^2+^), as a bridging link for various proteins, can competitively preempt zinc transporter 1 (ZnT1) that mediate ex‐copper entry into cells, thereby blocking the continued uptake of ex‐copper.^[^ ^11^ ^]^ Based on this, zinc therapy is widely recognized as a prominent adjunctive treatment in the clinical management of WD.^[^ ^12^ ^]^ However, excessive Zn^2+^ can potentially induce mitochondrial dysfunction and thereby enhance endogenous ROS production, leading to certain toxic effects.^[^ ^13^ ^]^ Therefore, the secure implementation of zinc therapeutics via ion‐exchange technology constitutes a vital strategy for efficaciously blocking the uptake pathway of ex‐copper, but it poses significant challenges.

Among the various potential ion exchange agents, curcumin (Cur), a representative natural antioxidant, has been reported to exhibit coordination with manganese ion, Cu^2+^, Zn^2+^ and other metal ions due to its highly conjugated *β*‐dione group, thereby demonstrating selectivity toward specific metal ions.^[^ ^14^, ^15^ ^]^ Interestingly, the solubility of Cur is significantly increased upon chelation with Zn^2+^, while its antioxidant property is markedly enhanced following chelation with Cu^2+^. Most importantly, the higher stability constant of copper‐cur (Cu‐Cur) compared to zinc‐cur (Zn‐Cur) (*K*_Cu‐Cur_ = 3.4 × 10^14^ mol^−1^, *K*_Zn‐Cur_ = 9 × 10^10^ mol^−1^), makes Zn‐Cur as a suitable ion exchange agent for displacing Cu^2+^ to form the stable Cu‐Cur and free Zn^2+^.^[^ ^16^ ^]^ Based on this, we hypothesize that if the ion‐exchange reaction occurs specifically in the damaged GnRH neurons of WD, it would enable the in situ synthesis of Cu‐Cur for reducing in‐copper and anti‐inflammatory, and releases free Zn^2+^ to competitively occupy with ZnT1 protein for blocking the uptake pathway of ex‐copper.

**Scheme 1:**
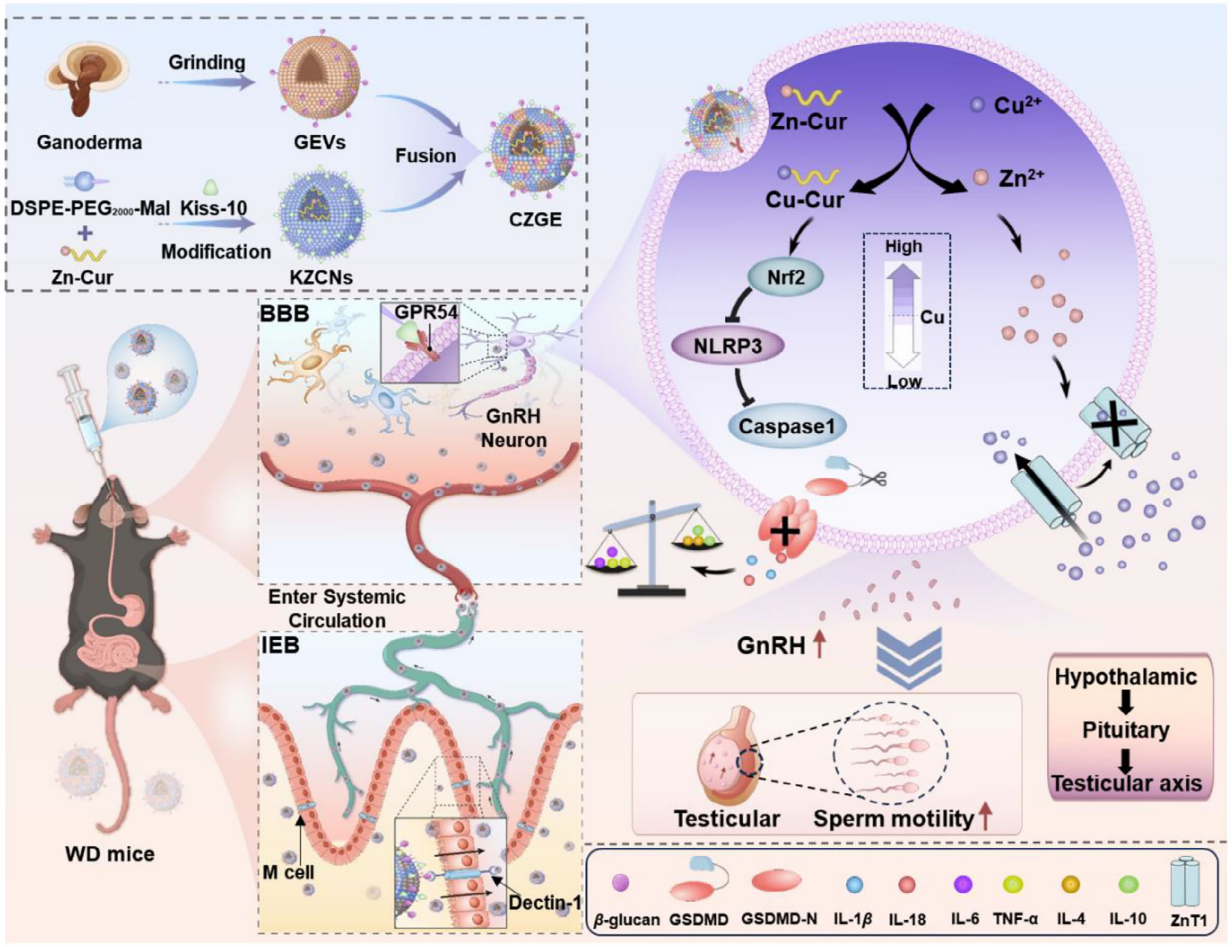
Schematic illustration of engineered Ganoderma‐derived extracellular vesicle synthesis and its application in the treatment of WD with reproductive dysfunction. Fusion of GEVs enriched *β*‐glucan extracted from the ganoderma and KZCNs prepared with self‐assembling technique to obtain hybrid CZGE. After oral intragastric administration, GEVs rich in *β*‐glucan specifically penetrated the IEB and BBB. Meanwhile, the Kiss‐10 peptide immobilized on the surface of CZGE was recognized and captured by the GPR54 receptor of GnRH neurons. The Zn‐Cur released in the high intracellular copper environment was subsequently replaced by Cu‐Cur and free Zn^2+^. The former can effectively reduce in‐copper and neuroinflammation through the Nrf2‐NLRP3 pathway, and the latter can block ex‐copper influx through activation of ZnT1. Consequently, it promoted the hormone secretion function of GnRH neurons, thus restoring reproductive dysfunction via the HPT axis in WD model mouse.

Here, an oral engineered Ganoderma‐derived extracellular vesicles (CZGE) was proposed based on ion exchange strategy for WD with reproductive dysfunction therapy. Specifically, Ganoderma‐derived extracellular vesicles enriched with *β*‐glucan (GEVs) were fused with Zn‐Cur nanomicelles modified with targeting peptides of kisspeptin‐10 (KZCNs). With the help of the penetrating ability of *β*‐glucan on GEVs, GZGE first penetrated the intestinal epithelial barrier (IEB) and blood brain barrier (BBB) through specific binding to M cells. After entering the brain parenchyma, efficient endocytosis of GnRH neurons is facilitated through kisspeptin‐10 (Kiss‐10) on ZCNs specific binding to G Protein‐Coupled Receptor 54 (GPR54) protein on the surface of GnRH neurons. The Zn‐Cur released in the high in‐copper environment was subsequently replaced by Cu‐Cur and free Zn^2+^. On one hand, the formation of Cu‐Cur effectively decreased in‐copper and augmented antioxidant activity. On the other hand, the presence of free Zn^2+^ competitively occupy with ZnT1 protein, thereby blocking the uptake pathway of ex‐copper. Consequently, it promoted the hormone secretion function of GnRH neurons, thus restoring reproductive dysfunction via the HPT axis in WD model mouse. The findings of our study demonstrated a novel nanomedicine that employed an ion‐exchange strategy, thereby facilitating a dual regulation approach involving in‐copper chelation and ex‐copper blockage to effectively enhance the treatment of WD accompanied by reproductive dysfunction (**Scheme** 1).

## 2 Results and Discussion

### 2.1 Clinical Indexes Analysis of WD with Reproductive Dysfunction

**Figure 1:**
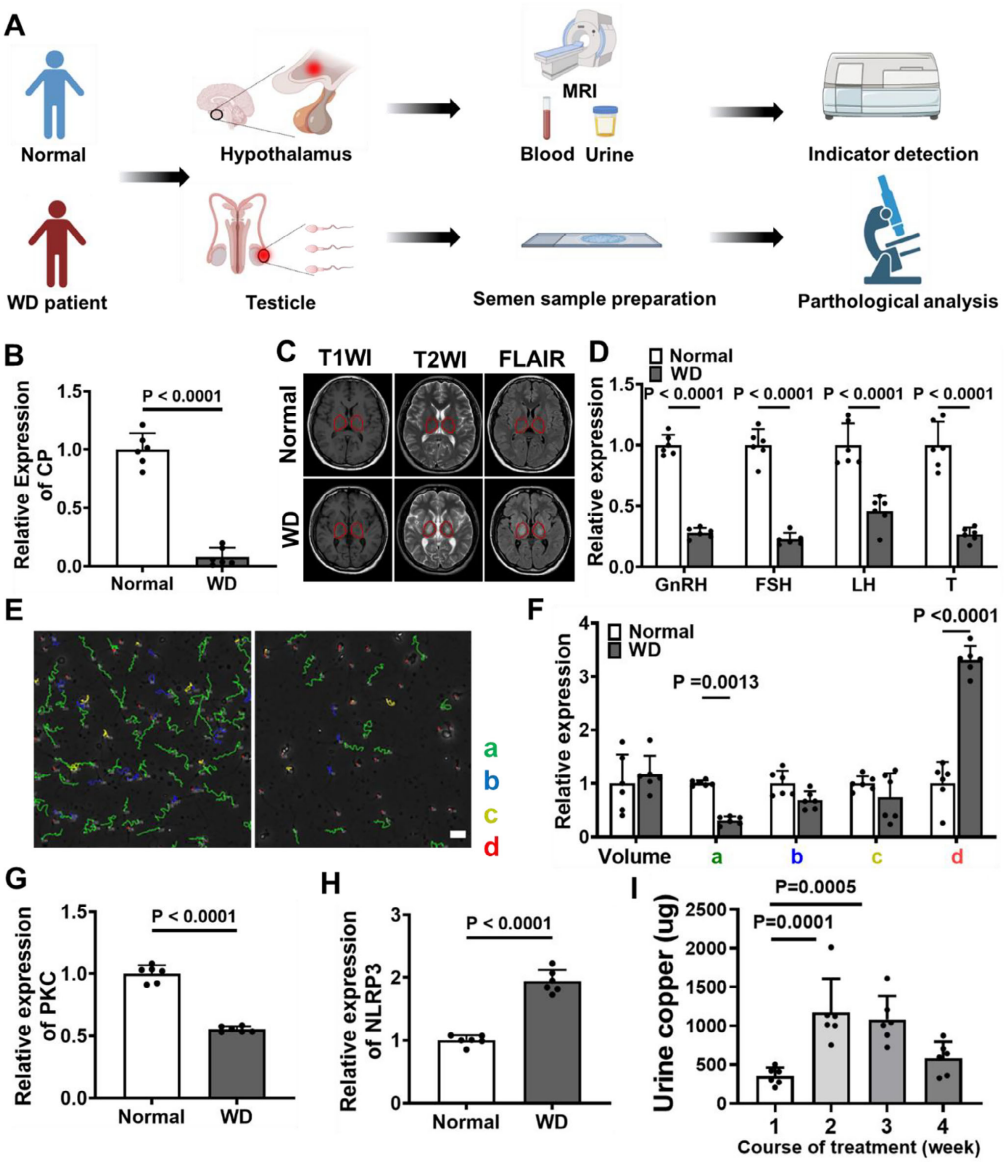
Clinical indexes analysis from patients WD with reproductive dysfunction (*n* = 6) and healthy donors (*n* = 6). A) Flowchart of the collection and analysis of clinical indicators. B) Relative quantitative analysis of CP in serum, in which the expression of normal group as denoted as 1. C) Transverse section MRI images of the brain. D) Relative expression of GnRH, FSH, LH and T in serum, in which the expression of normal group as denoted as 1. E) Sperm motility trajectory map, scale bar: 20 µm. F) Relative assessment of semen parameters. G,H) Relative quantitative analysis of NLRP3 (G) and PKC (H) in serum, in which the expression of normal group as denoted as 1. I) 24 h urine copper levels of the patients WD with reproductive dysfunction in different course of treatment. All statistics are expressed as mean ± standard deviation. Statistical analysis: one‐way ANOVA followed by Tukey's HSD post hoc test.

To address the current gap in the clinical application of nanotechnology‐based ion‐regulating agents for WD with reproductive dysfunction, the screening and identification of effective targets were initiated by healthy donors and WD with reproductive dysfunction patients in **Figure** **1**A. Ceruloplasmin (CP) and magnetic resonance imaging (MRI), as a common criterion for clinical diagnosis of WD, were used to evaluate the copper deposition in WD patients.^[^ ^17^, ^18^ ^]^ The level of CP in WD with reproductive dysfunction patients were markedly reduced compared to those observed in healthy donors (Figure 1B). Besides, WD with reproductive dysfunction patients exhibited distinct MRI imaging characteristics compared to the healthy donors. Specifically, the symmetric hypointense on T1‐weighted images (T1WI) while the slightly hyperintense on T2‐weighted images (T2WI) and fluid‐attenuated inversion recovery (FLAIR) sequences, were clearly observed in the brain MRI (Figure 1C). This finding was indicative of potential copper deposition in the brain.

Theoretically, excessive copper accumulation in the hypothalamus of WD patients, specifically within GnRH neurons, may lead to impaired GnRH secretion, consequently decreasing the levels of GnRH in blood.^[^ ^19^ ^]^ It could in turn affected the synthesis and secretion of follicle‐stimulating hormone (FSH), luteinizing hormone (LH), and testosterone (T) through the cascade regulation of the HPT axis.^[^ ^20^ ^]^ It was evidenced that the levels of GnRH, FSH, LH, and T in serum of WD with reproductive dysfunction patients were found to be reduced by 3.57, 4.39, 2.19, and 3.73 times than that in healthy donors, respectively (Figure 1D). In the evaluation of sperm function in WD with reproductive dysfunction patients, a comprehensive semen analysis was performed to assess sperm motility^[^ ^21^ ^]^ In comparison to sperm from healthy donors, the proportion of grade a + b sperm (Grade a > b > c > d) in these WD with reproductive dysfunction patients was significantly reduced to 28.95%, whereas the proportion of graded sperm was notably elevated Figure 1E,F. This observation suggested a substantial increase in sperm immotility within these patients, which manifests clinically as asthenospermia.

Previous studies have reported that dysregulation of copper homeostasis in the brain may activate the nucleotide‐binding domain and leucine‐rich repeat family pyrin domain containing 3 (NLRP3) ‐associated pyroptosis pathway, which was implicated in the apoptosis of neurons.^[^ ^22^ ^]^ In light of this, an investigation was conducted to ascertain the presence of pyroptosis in WD with reproductive dysfunction patients. As shown in Figure 1G,H, the level of NLRP3 was significantly elevated while the level of protein kinase C (PKC) were notably decreased in WD with reproductive dysfunction patients compared to those healthy donors. The observed outcome supported a hypothesis that excess copper may modulate the NLRP3‐PKC pathway, thereby influencing the function of GnRH neurons. Zinc agents, as first‐line therapeutics in the clinical management of WD, had demonstrated efficacy in inhibiting ex‐copper absorption and enhancing its excretion.^[^ ^23^ ^]^ To further elucidate the therapy role of zinc agents in WD with reproductive dysfunction patients, the copper excretion in urine significantly increased during the treatment period, subsequently declined, and eventually stabilized, which confirmed that zinc agents could inhibit the absorption of ex‐copper (Figure 1I). On the basis of the above clinical experiments results, the effectively removal of ex‐copper by zinc agents and the reduction of in‐copper by modulating the NLRP3‐PKC pathway can significantly reshape copper metabolism for WD with reproductive dysfunction therapy. Therefore, an oral engineered extracellular vesicle based on ion exchange strategy was designed in the subsequent experiment, thereby involving in‐copper chelation and ex‐copper blockage.

### 2.2 Synthesis and Characterization of CZGE

**Figure 2:**
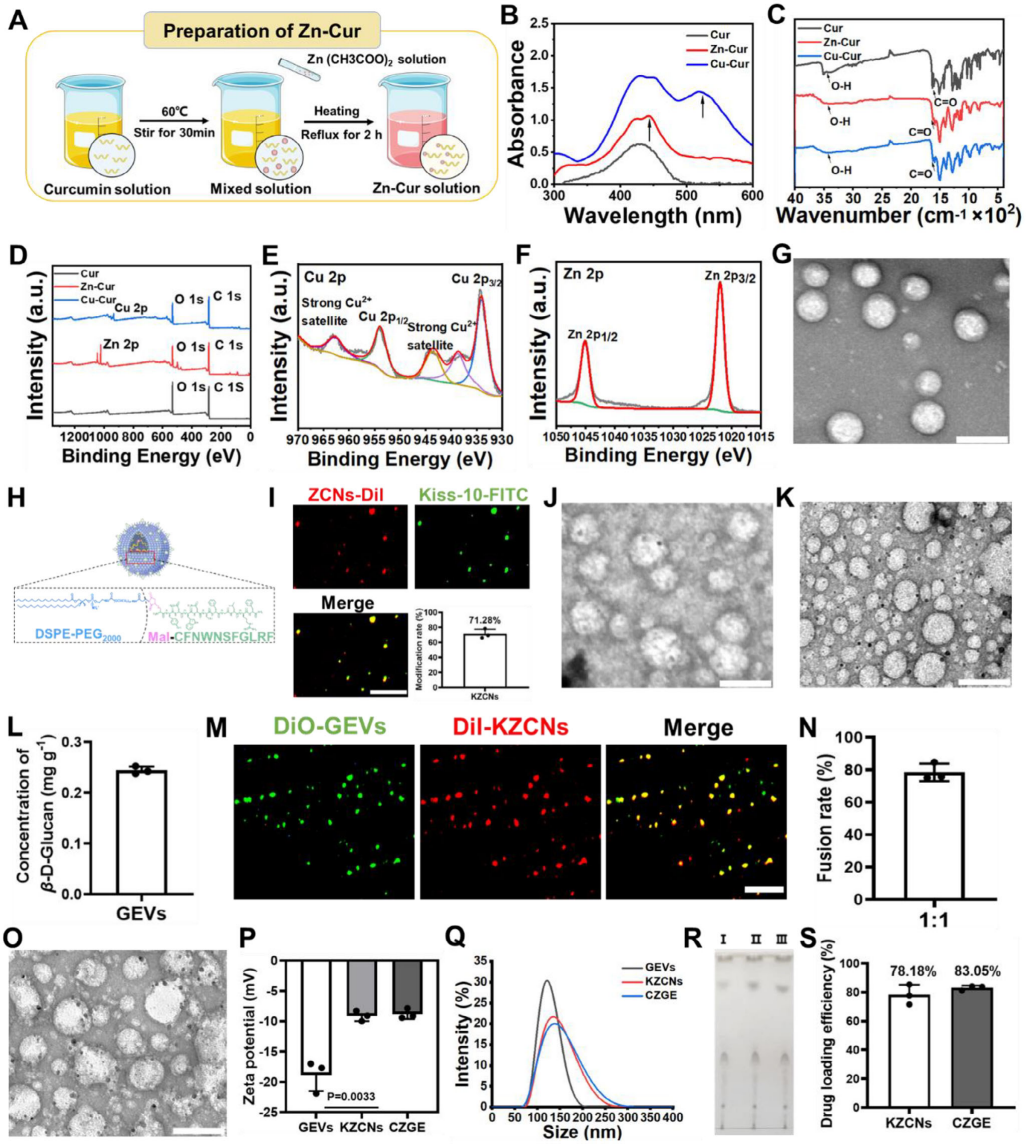
Synthesis and characterization of CZGE. A) Schematic diagram of the preparation of Zn‐Cur. B) The UV–vis absorption spectra of Cur, Zn‐Cur, and Cu‐Cur. C) The FTIR spectra of Cur, Zn‐Cur, and Cu‐Cur. D) The XPS spectrum of Cur, Zn‐Cur, and Cu‐Cur. E,F) XPS spectra of Cu 2p in Cu‐Cur (E) and Zn 2p in Zn‐Cur (F). G) TEM image of ZCNs, scale bar: 200 nm. H) The surface structure of KZCNs. I) Fluorescence images of KZCNs with Dil labeled DSPE‐PEG‐Mal (red) and FITC labeled Kiss‐10 (green) and modification rate of Kiss‐10 on KZCNs (*n* = 3), scale bar: 500 nm. J) TEM image of KZCNs, scale bar: 200 nm. K) TEM image of GEVs, scale bar: 200 nm. L) The concentration of *β*‐glucan in GEVs (*n* = 3). M, N) Fluorescence images of CZGE with 1:1 ratio of KZCNs (red) to GEVs (green) (M) and fusion rate (N). O) TEM image of CZGE, scale bar: 200 nm. P,Q) Zeta potential (P) and hydrodynamic diameters (Q) of different samples (*n* = 3). R) The thin layer chromatography analysis of different samples (I: Ganoderma; II: GEVs; III: CZGE). S) Drug loading efficiency of different samples (*n* = 3). All statistical data are presented as mean ± standard deviation. Statistical analysis: one‐way ANOVA followed by Tukey's HSD post hoc test.

Based on the aforementioned findings, it is imperative to design a targeted nanoformulations capable of dual regulation in‐copper chelation and ex‐copper blockade for WD with reproductive dysfunction therapy. An ion exchange agents (Zn‐Cur) was first prepared via the coordination method in **Figure** **2**A.^[^ ^24^ ^]^ The powders of Cur, Zn‐Cur and Cu‐Cur showed yellow, red and dark brown colors, such distinct color variations arise from the altered electronic structure resulting from metal‐ligand coordination (Figure S1, Supporting Information). Compared to that of Cur and Cu‐Cur, Zn‐Cur demonstrated superior dispersibility in aqueous solution, which facilitated its subsequent loading into nanocarriers (Figure S2, Supporting Information). Besides, the results of the ultraviolet‐visible (UV–vis) absorption spectrum displayed, a slight absorption peak at ≈445 nm emerged in the Zn‐Cur spectrum,^[^ ^8^ ^]^ while a distinct broad peak at 525 nm was observed in the Cu‐Cur spectrum, indicating to a stronger coordination between Cur and Cu^2+^ than between Cur and Zn^2+^ (Figure 2B).^[^ ^25^, ^26^ ^]^ This finding was further substantiated by the results obtained from fourier transform infrared (FTIR) spectroscopic analysis and ^1^H nuclear magnetic resonance spectra (^1^H NMR) (Figure 2C, Figure S3, Supporting Information).^[^ ^27^, ^28^, ^29^ ^]^ The X‐ray photoelectron spectroscopy (XPS) confirmed that the high‐resolution Cu 2p spectrum of Cu‐Cur exhibited two characteristic peaks at 934.5 eV (Cu 2p_3/2_) and 954.2 eV (Cu 2p_1/2_), accompanied by two oscillating satellite peaks, indicating that the presence of Cu^2+^ in the chelate complex. The high‐resolution Zn 2p spectrum of Zn‐Cur displayed two characteristic peaks at 1022 eV (Zn 2p_3/2_) and 1045.2 eV (Zn 2p_1/2_) (Figure 2D–F). Besides, an uniform spherical structures with an average diameter of 50 nm of Zn‐Cur was observed in transmission electron microscopy (TEM) image (Figure S4, Supporting Information).

After that, Zn‐Cur nanomicelles (ZCNs) was synthesized by the thin film dispersion technique,^[^ ^30^ ^]^ finding that a homogeneous circular dispersed nanosphere by TEM in Figure 2G. Kiss‐10 peptide with targeting ability to GPR54 protein on GnRH neurons was further synthesized and verified to conjugate to maleimide group of ZCNs by high‐performance liquid chromatography (HPLC) and time of flight (TOF) mass (Figure 2H and Figure S5, Supporting Information). It was found that 71.28% of Kiss‐10 peptide was grafted into ZCNs, named as KZCNs (Figure 2I,J). KCZNs exhibited a diameter of ≈125.4 nm (Figure S6, Supporting Information). And the zeta potential of KCZNs reached −9.13 mV, less negative potential than of ZCNs alone (−19.3 mV), which may be due to the modification of the positive potential of Kiss‐10 peptide on the ZCNs (Figure S7, Supporting Information). Besides, GEVs were extracted from fresh Ganoderma by the classic ultracentrifugation method, which was rich in *β*‐glucan (0.244 mg·g^−1^) (Figure 2K,L). Finally, GEVs was incorporated into the KZCNs to synthesize CZGE by membrane fusion technology. The successful fusion of GEVs (green) with KZCNs (red) was confirmed by fluorescence colocalization, resulting the highest fusion rate (78.49%) at a ratio of 1:1 and the förster resonance energy transfer (FRET) effect was further evaluated to confirm the synthetic CZGE by fusing GEVs and KZCNs (Figure 2M,N and Figure S8, Supporting Information). According to TEM, dynamic light scattering (DLS), nanoparticle tracking analysis (NTA) and zeta potentials measurements, the diameter of CZGE was ≈143.4 nm and displayed a negative potential of 8.8mV (Figure 2O–Q and Figure S6, Supporting Information). Next, we determined the effects of the fusion process on the surface proteins of GEVs and Zn‐Cur loading. It was found that most of the protein was retained and the Zn‐Cur loading was still 78.18% (Figure 2R,S and Figure S9, Supporting Information). Additionally, the stability of various samples was evaluated by tracking their particle size changes throughout a 7‐day observation period. There was no apparent aggregation‐induced precipitation, indicating the satisfactory stability for long term application (Figure S10, Supporting Information). These findings demonstrated that the modification of *β*‐glucan and encapsulation of Zn‐Cur have been successfully prepared in CZGE.

### 2.3 Penetration and Ion Exchange Efficiency of CZGE In Vitro

**Figure 3:**
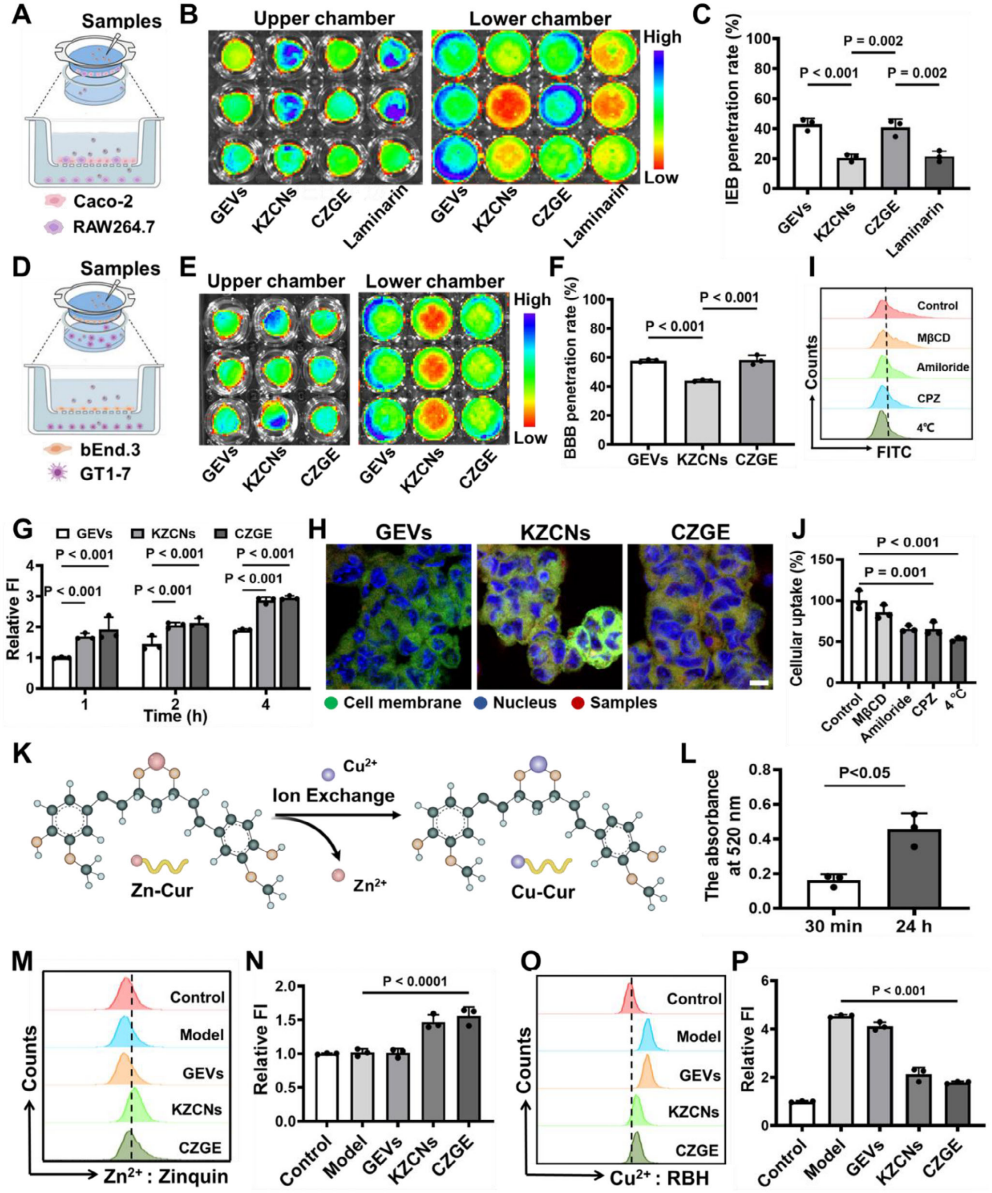
Penetration and ion exchange efficiency of CZGE in vitro. A) Schematic representation of the in vitro construction process for IEB. B) Fluorescence images of the upper chamber and lower chambers incubation of different Cy5 labeled‐samples using an in vivo imaging system (IVIS). C) IEB penetration rate of different Cy5 labeled‐samples (*n* = 3). D) Schematic representation of the in vitro construction process for BBB. E) Fluorescence images of the upper chamber and lower chambers incubation of different Cy5 labeled‐samples using an in vivo imaging system (IVIS). F) BBB penetration rate of different Cy5 labeled‐samples (*n* = 3). G) Relative FI of different samples after different cocultured time by flow cytometry (*n* = 3). H) Representative co‐localization CLSM images of different samples (red), cell membrane (green) and nucleus (blue), scale bar: 20 µm. I,J) The uptake mechanism of CZGE under different condition by flow cytometry (*n* = 3). K) Scheme of Cu‐Cur formation and Zn^2+^ release after nanomedicine degradation through an ion‐exchange reaction. L) The intracellular Cu‐Cur levels investigation of GT1‐7 cells after CZGE treatment by UV–vis absorption spectra (*n* = 3). M,N) The intracellular Zn^2+^ levels investigation of GT1‐7 cells under different conditions stained with Zn^2+^ fluorescence probe detected by flow cytometry, in which the FI of Zn^2+^ in the control group was denoted as 1 (*n* = 3). O,P) The intracellular Cu^2+^ levels investigation of GT1‐7 cells under different conditions stained with Cu^2+^ fluorescence probe detected by flow cytometry, in which the FI of Cu^2+^ in the control group was denoted as 1 (*n* = 3). All statistical data are presented as mean ± standard deviation. Statistical analysis: one‐way ANOVA (C, F, J, L, N, P); two‐way ANOVA (G).

To align with the currently prevalent oral treatment methods for WD in clinical practice, a critical prerequisite for the efficacy of the CZGE with abundant of *β*‐glucan proposed in this work is its ability to successively penetrate the IEB and BBB.^[^ ^31^, ^32^, ^33^ ^]^ Prior to evaluating the penetration, the optimal concentration (10^6^ particles mL^−1^) and incubation time (24 h) for CZGE to regulate the activity of GnRH neurons were determine for subsequent experiments (Figure S11, Supporting Information). First, human colorectal adenocarcinoma cells (Caco‐2) and mouse mononuclear macrophage leukemia cells (RAW264.7) were seeded in the upper and lower chamber by transwell model to simulate the IEB in vitro (**Figure** **3**A–C). The IEB penetration efficiency of GEVs and CZGE was 42.87% and 40.89%, respectively, which was 2.08 and 1.99 times higher than that of KZCNs. This difference indicated the important role of *β*‐glucans in penetrating the IEB. To further elucidated the mechanism by which *β*‐glucan penetrates intestinal M cells through specific recognition by the Dectin‐1 receptor, laminarin, a specific inhibitor of Dectin‐1, was utilized to pre‐treat IEB co‐culture models. Subsequently, the penetration rates of different samples were assessed, and the penetration rate of CZGE was found to be significantly reduced to 21.15%, as anticipated. In addition, the cellular uptake of different samples by the lower chamber was further observed (Figure S12, Supporting Information). The fluorescence intensity of the CZGE group was the strongest compared to that in other group, indicating that it had better intracellular internalization. Subsequently, a co‐culture model of mouse brain‐derived Endothelial cells.3 (bEnd.3) and mouse hypothalamic neuron (GT1‐7) was established to simulate the BBB in vitro (Figure 3D,E). As shown in Figure 3F, the penetration efficiency of the BBB by GEVs and CZGE was 57.69% and 58.19%, respectively, which was 1.31 and 1.32 times higher than that of KZCNs. Additionally, fluorescence images of GTI‐7 cells in the lower chamber treated by different samples were also investigated in Figure S13, Supporting Information. It indicated that CZGE successfully penetrated the BBB model and was taken up by GTI‐7 cells in the lower chamber.

Theoretically, the GPR54 protein on the membrane surface of GnRH neurons is specifically bound by Kiss‐10 peptide, thereby targeting GnRH neurons.^[^ ^34^ ^]^ Flow cytometry analysis revealed a time‐dependent uptake of different Cy5‐labeled samples in GT1‐7 cells, and the average fluorescence intensity of KZCNs and CZGE at 4 h was found to be significantly higher than that of GEVs (Figure 3G). The finding was in line with the observed trend of qualitative analysis, indicating that the extensive uptake of CZGE by GT1‐7 cells can be enhanced through the action of the Kiss‐10 peptide (Figure 3H and Figure S14, Supporting Information). Subsequently, the mechanism of CZGE uptake by GT1‐7 cells was further investigated (Figure 3I,J). First, low temperature treatment at 4 °C significantly inhibited the efficiency of cellular uptake of CZGE by GT1‐7 cells, indicating that the uptake mechanism was dependent on energy endocytosis. Additionally, only in the presence of methyl‐*β*‐cyclodextrin (M*β*CD, a caveolin‐mediated inhibitor), the uptake efficiency of CZGE in GT1‐7 cells was found to be minimally affected, indicating that CZGE was dependent on clathrin‐mediated and macropinocytosis mediated uptake pathways. The results indicated that GT1‐7 cells may consume more energy to uptake CZGE through the three energy‐dependent intakes pathways mentioned above.

We assume that the Zn‐Cur released from CZGE will undergo ion exchange reaction with excess intracellular Cu^2+^ to displace both Zn^2+^ and Cu‐Cur (Figure 3K). Here, analysis of intracellular Cu‐Cur, Cu^2+^ and Zn^2+^ level was performed to verify the effectiveness of ion exchange strategy by CZGE using CuSO_4_‐induced WD copper‐overloaded cell model in vitro. The level of Cu‐Cur in GT1‐7 cells following CZGE treatment was detected using UV–vis absorption spectrum. The characteristic peak intensity of Cu‐Cur at 520 nm was observed to significantly increase after 24 h, indicating that CZGE can effectively generate Cu‐Cur in GT1‐7 cells of the model group (Figure 3L). Then, the zinquin ethyl ester fluorescence probe was further used to evaluate the effects of different samples on the Zn^2+^ level in GT1‐7 cells of the model group. It showed that the Zn^2+^ level in the cells was significantly increased after KZCNs and CZGE treatment (Figure 3M,N). Finally, a significant increase in Cu^2+^ level was observed in the model group compared to the control group. However, after KZCNs and CZGE treatment, there was a reduction in Cu^2+^ level (Figure 3O,P and Figure S15, Supporting Information). The above results indicated that Zn^2+^ was effectively replaced by CZGE by chelating intracellular Cu^2+^, thereby enhancing the subsequent treatment efficacy and minimizing potential side effects.

### 2.4 The Therapy Mechanism of CZGE In Vitro by Ion Exchange Strategy

**Figure 4:**
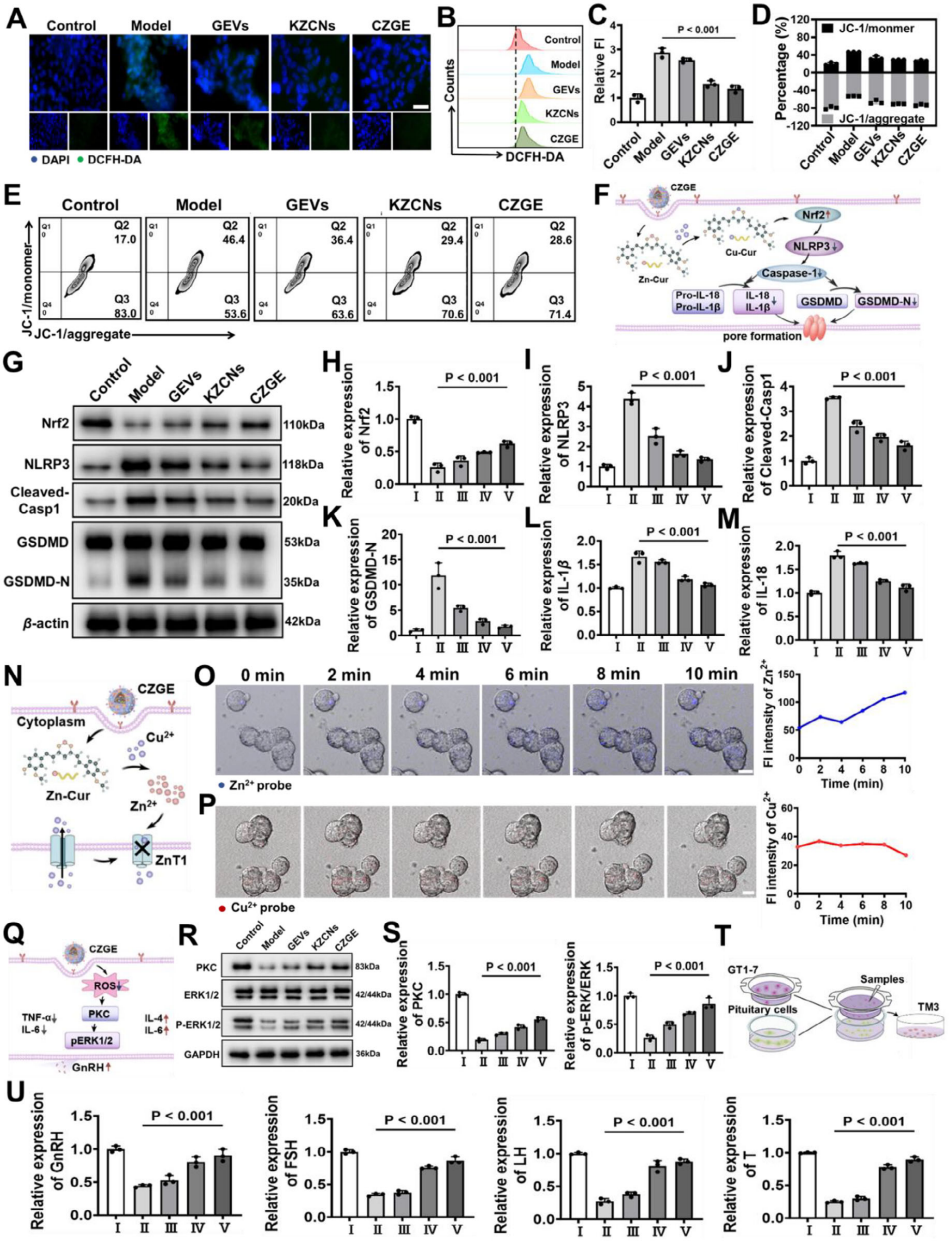
The therapy mechanism of CZGE in vitro by ion exchange strategy. A) Representative fluorescent images of GT1‐7 cells treated with different samples (green: ROS fluorescence probe, blue: nucleus), scale bar: 50 µm. B,C) The elimination of ROS in GT1‐7 cells under different conditions stained with ROS fluorescent probes by flow cytometry, in which the FI of ROS in the control group was denoted as 1 (*n* = 3). D,E) Quantitative determination of mitochondrial membrane potentials after different treatments by flow cytometry (*n* = 3). F) Schematic illustration on mechanism of pyroptosis regulation by CZGE throughout the process. G–K) Representative western blot image and quantification of the relative protein expression of Nrf2, NLRP3, cleaved caspase‐1, and GSDMD‐N (*n* = 3). L,M) Relative quantification of IL‐18 (L) and IL‐1*β* (M) in GT1‐7 cells by different treatments (*n* = 3). N) Schematic illustration on mechanism of inhibiting ex‐copper entry by CZGE throughout the process. O) Time‐course fluorescence microscopy images and quantification of GT1‐7 cells after incubation with Zn^2+^ fluorescent probe (blue) and addition of CZGE, scale bar: 5 µm. P) Time‐course fluorescence microscopy images and quantification of GT1‐7 cells after incubation with CZGE, Cu^2+^ fluorescent probe (red) and another addition of CuSO_4_, scale bar: 10 µm. Q) Schematic illustration on mechanism of GnRH secretion through PKC/ERK pathway by CZGE throughout the process. R,S) Representative western blot image and quantification of the relative protein expression of PKC, ERK1/2 and p‐ERK1/2 (*n* = 3). T) Schematic illustration of the co‐culture. U) Relative quantification of GnRH, FSH, LH, T in GT1‐7 cells by different treatments, in which the expression of control group was denoted as 1 (*n* = 3). (Samples for H‐U: I: control, II: model, III: GEVs, IV: KZCNs, V: CZGE). All statistical data are presented as mean ± standard deviation. Statistical analysis: one‐way ANOVA followed by Tukey's HSD post hoc test.

Following confirmation that CZGE entered cells and generated Cu‐Cur and Zn^2+^ via ion exchange and effectively reduced the intracellular Cu^2+^ level, we further explored its ion regulation mechanism. As a classic metal‐polyphenol chelate, Cu‐Cur has been reported to efficiently catalyze ROS through phenol groups and was employed in the treatment of diseases (sunch as osteoarthritis).^[^ ^8^ ^]^ Using electron spin resonance (ESR) spectroscopy, we observed significant weakened of the characteristic signal O_2_^• −^ (1:1:1:1) upon Cu‐Cur addition, demonstrating its effective O_2_^• −^ scavenging ability, consistent with SOD activity kit results. Similar ^•^OH scavenging effects were observed (Figure S16, Supporting Information).^[^ ^35^ ^]^ Subsequently, the ROS clearance effects of different samples on copper‐overloaded GT1‐7 cells were further evaluated using 2,7‐dichlorodihydrofluorescein diacetate (DCFH‐DA) fluorescent probes (**Figure** **4**A–C). The ROS levels in GT1‐7 cells of the model group were found to be significantly elevated, whereas they were significantly reduced following intervention with KZCNs and CZGE. The above findings suggested that the incorporation of Cu‐Cur in cells effectively replaced the loaded Zn‐Cur in KZCNs and CZGE, leading to a decrease in intracellular ROS levels. Oxidative stress often induces mitochondrial dysfunction, which can directly or indirectly trigger the release of inflammatory factors, ultimately leading to cellular damage or apoptosis.^[^ ^36^ ^]^ As anticipated, flow cytometry revealed that the ratio of Tetraethyl benzimidazolyl carbocyanine iodide (JC‐1) aggregates (indicative of healthy mitochondria) to JC‐1 monomers (indicative of mitochondria with impaired membrane potential) was significantly increased in the model group following treatment with CZGE. This effectively counteracted mitochondrial damage (Figure 4D,E). Moreover, the results of ELISA kit indicated that the levels of intracellular anti‐inflammatory factors (such as interleukin‐10 (IL‐10) and interleukin‐4 (IL‐4)) were significantly elevated following CZGE treatment, while the levels of pro‐inflammatory factors (such as interleukin‐6 (IL‐6) and tumor necrosis factor α (TNF‐α)) were significantly decreased (Figure S17, Supporting Information). Subsequently, the effects of different samples on the polarization phenotype of BV2 cells were assessed using in vitro flow cytometry. The results demonstrated that in the model group, the expression of the M1 phenotype marker (CD86) was elevated, while the expression of the M2 phenotype marker (CD206) was reduced. Following treatment with GEVs and KZCNs, the CD86 level in BV2 cells was decreased to 12.1% and 8.87%, respectively, and the CD206 level was increased to 4.05% and 22.7%, respectively. Upon intervention with CZGE, the CD206 level was further elevated to 27.2%. It indicated that CZGE can promote the transformation of BV2 cells toward an anti‐inflammatory M2 phenotype (Figure S18, Supporting Information). These results further substantiated that oxidative stress induced by exposure of GnRH neurons to copper could be significantly reduced by CZGE, that neuroinflammation could be inhibited, and that the brain microenvironment could be improved.

Furthermore, it has been reported that dysregulation of copper homeostasis in the brain can facilitate the assembly of the NLRP3 inflammasome and induce neuronal damage via pyroptosis, which may subsequently affect GnRH secretion and contribute to reproductive dysfunction.^[^ ^37^ ^]^ To investigate the correlation between CZGE anti‐inflammation and pyroptosis, the expression levels of related proteins involved in pyroptosis were further examined by western blotting (Figure 4G–K). Compared with that in the model group, significantly up‐regulated expression of nuclear factor erythroid 2‐related factor 2 (Nrf2) while down‐regulated expression of NLRP3, cleaved Caspase‐1 and gasdermin D N‐terminal domain (GSDMD‐N) were observed in the CZGE group. Besides, interleukin‐1*β* (IL‐1*β*) and interleukin‐18 (IL‐18) play a key role in inflammasome activation and cell pyrodeath.^[^ ^38^, ^39^, ^40^, ^41^ ^]^ The expression levels of IL‐1*β* and IL‐18 was significantly reduced after CZGE treatment (Figure 4L,M). Then, we subjected the cells to treatment with the NLRP3 inhibitor (MCC950) and observed that the level of NLRP3 in the model group cells was 2.75 times higher than that in the NLRP3 inhibitor group, indicating that excess Cu^2+^ can promote the expression of NLRP3. Furthermore, the level of NLRP3 in the cells treated by CZGE also decreased significantly, suggesting that CZGE can inhibit the expression of NLRP3 by reducing copper deposition (Figure S19A, Supporting Informing). Conversely, the PKC level in cells of the NLRP3 inhibitor group was significantly higher than that in the model group, a finding consistent with the PKC trend in the CZGE group (Figure S19B, Supporting Informing). These results indicated that copper deposition can affect GnRH secretion by regulating the NLRP3‐PKC pathway. Based on the above results, it was plausible to infer that Cu‐Cur produced through the intracellular ion exchange process in CZGE, can effectively attenuate inflammation by inhibiting the NLRP3/Caspase‐1/GSDMD pyroptosis signaling pathway (Figure 4F).

Ex‐copper serves as a critical source of in‐copper, and its effective inhibition is crucial for the long‐term management of WD with reproductive dysfunction. Previous research have demonstrated that the continuous influx of ex‐copper into cells can be inhibited by Zn^2+^ through the modulation of ZnT1 (Figure 4N). Building on this foundation, fluorescent probes were employed in this study to monitor real‐time changes in Zn^2+^ and Cu^2+^ levels in the model group treated with CZGE, with the aim of elucidating the effects of CZGE on the balance of Zn^2+^ and Cu^2+^ inside and outside the cells and its underlying mechanisms. Initially, confocal real‐time imaging using a Zn^2+^ fluorescent probe revealed a significant enhancement in cellular Zn^2+^ fluorescence intensity within 10 min of CZGE addition, indicating an increase in intracellular Zn^2+^ levels (Figure 4O). Moreover, WB analysis revealed that the expression level of ZnT1 significantly increased following CZGE treatment compared with that in the model group. This increase might be attributed to the replacement of in‐copper by CZGE in a high‐copper environment, thereby causing an elevation in the intracellular free Zn^2+^ level (Figure S20, Supporting Information). Furthermore, following a 4 h pretreatment with CZGE and subsequent addition of CuSO_4_, no significant change in intracellular Cu^2+^ fluorescence intensity was observed, suggesting that Zn^2+^ significantly inhibits the entry of ex‐copper into cells (Figure 4P). In contrast, the model cells without CZGE pretreatment exhibited a gradual increase in Cu^2+^ fluorescence intensity 10 min after the addition of CuSO_4_, further confirming the specificity and efficacy of Zn^2+^ in blocking copper influx (Figure S21, Supporting Information).

Subsequently, a similar preparation method was used to synthesize CZGE excepting for Cur (named CZGE*), to further verify the effect of Zn^2+^ on in‐copper and ex‐copper copper levels (Figure S22, Supporting Information). The flow cytometry results showed that the Cu^2+^ level of cells in the model group with another addition of CuSO_4_ (II group) was significantly increased compared with that in the model group (I group), indicating that the continuous inflow of ex‐copper can increase the level of in‐copper. In contrast, no significant increase in the Cu^2+^ level of cells was observed after pre‐incubation with CZGE* and subsequent additional CuSO4 stimulation (III group), indicating that Zn^2+^ could inhibit the inflow of ex‐copper. Besides, the cells were pre‐treated with ZnT1 gene siRNA (si‐ZnT1) to inhibit the expression of ZnT1 protein. The quantitative real‐time polymerase chain reaction (RT‐qPCR) test confirmed that the expression of ZnT1 was decreased in the si‐ZnT1 groups. Therefore, the si‐ZnT1 was selected for subsequent experiments (Figure S23, Supporting Information). As illustrated in Figure S22, Supporting Information, despite the inhibition of ZnT1 expression by si‐ZnT1 in cells, the Cu^2+^ levels in cells treated with si‐ZnT1 and CZGE* (IV group) were comparable to those of the model group (I group). This may be attributed to the minimal impact of ZnT1 on in‐copper levels. However, upon another addition of CuSO_4_ solution (V group), the Cu^2+^ levels in the cells significantly increased. This was attributed to the inhibition of ZnT1, which could not prevent the influx of external copper, thereby resulting in a significant elevation in cellular Cu^2+^ levels. Furthermore, a similar preparation method was employed to synthesize CZGE lacking Zn^2+^ but with the same other factors (named CZGE**). The investigation focused on whether Cur, another key component of CZGE, mediates the regulation of ZnT1 on in‐copper and ex‐copper levels (Figure S24, Supporting Information). Even when the protein expression of ZnT1 was blocked in si‐ZnT1 pretreated cells, the Cu^2+^ level in the cells treated with CZGE** (III group) was still lower than that of the model group (I group). Nevertheless, upon re‐addition of CuSO_4_ (simulating continuous ex‐copper influx), the Cu^2+^ level in CZGE**‐treated cells was maintained at a high level. This indicated that while the chelation of Cu^2+^ by Cur effectively reduced in‐copper levels, it had difficulty in preventing the influx of ex‐copper. The above results indicated Cur and Zn^2+^ as the two key components were released from CZGE upon cell entry. The former reduced the in‐copper level through chelation, while the latter acted on the membrane‐localized ion channel ZnT1 to inhibit the continuous influx of ex‐copper.

As an upstream hormone affecting T secretion, GnRH was regulated by PKC/ERK pathway, which influenced GnRH secretion by regulating the depolarization of GnRH neurons (Figure 4Q).^[^ ^42^ ^]^ As depicted in Figure 4R,S, the phosphorylation levels of PKC protein and ERK1/2 in the model group were significantly down‐regulated, contributing to abnormal GnRH secretion due to oxidative damage induced by copper overload. In contrast, the phosphorylation levels of PKC and ERK1/2 were significantly up‐regulated in cells treated with CZGE compared to the model group, indicating enhanced intracellular ROS scavenging. GT1‐7 cells and pituitary cells were co‐cultured in a 3D transwell chamber. The GnRH levels in upper chamber solution and FSH and LH levels in lower chamber solution were detected treatment with different samples. Additionally, the levels of T in TM3 cells was measured after 24 h of culture with lower chamber solution, in order to evaluate the potential of CZGE to improve reproductive function in vitro (Figure 4T). It was discovered that the levels of GnRH, FSH, LH, and T in the CZGE group were significantly higher than those in the model group (Figure 4U). This increase could be attributed to the abality of CZGE to prevent cell death caused by copper overload and to reduce oxidative stress. These actions led to the restoration of GnRH neuron endocrine function, regulation of the HPT axis, and promotion of reproductive hormone secretion, thereby improving reproductive function.

### 2.5 Evaluation of the Target Ability of CZGE In Vivo

To ensure the efficacy of CZGE as an oral nanomedicine, maintaining its stability in the gastrointestinal (GI) fluid environment is a critical prerequisite for therapeutic effectiveness. The stability of GEVs, KZCNs, and CZGE was evaluated in simulated gastric fluid (SGF, pH = 1.8) and simulated intestinal fluid (SIF, pH = 7.4) using DLS and subsequent drug release analysis. After 4 h of treatment in both SGF and SIF, the particle sizes of GEVs and CZGE remained comparable to those treated with KZCNs and PBS, respectively (Figure S25, Supporting Information). Additionally, the retention rate of Zn‐Cur in CZGE remained largely unchanged under these conditions, suggesting that CZGE exhibits stability in the GI tract (Figure S26, Supporting Information). It may be attributed to the protective structure of plant‐derived EVs with acid resistance.^[^ ^43^ ^]^ Besides, the blood circulation time of CZGE (half‐life of 4.12 h) in healthy Sprague‐Dawley rats was significantly prolonged than that (half‐life of 1.66 h) of free Cy5, which suggested that CZGE exhibits superior in vivo stability and biocompatibility, thereby extending its residence time within the body (Figure S27, Supporting Information).

**Figure 5:**
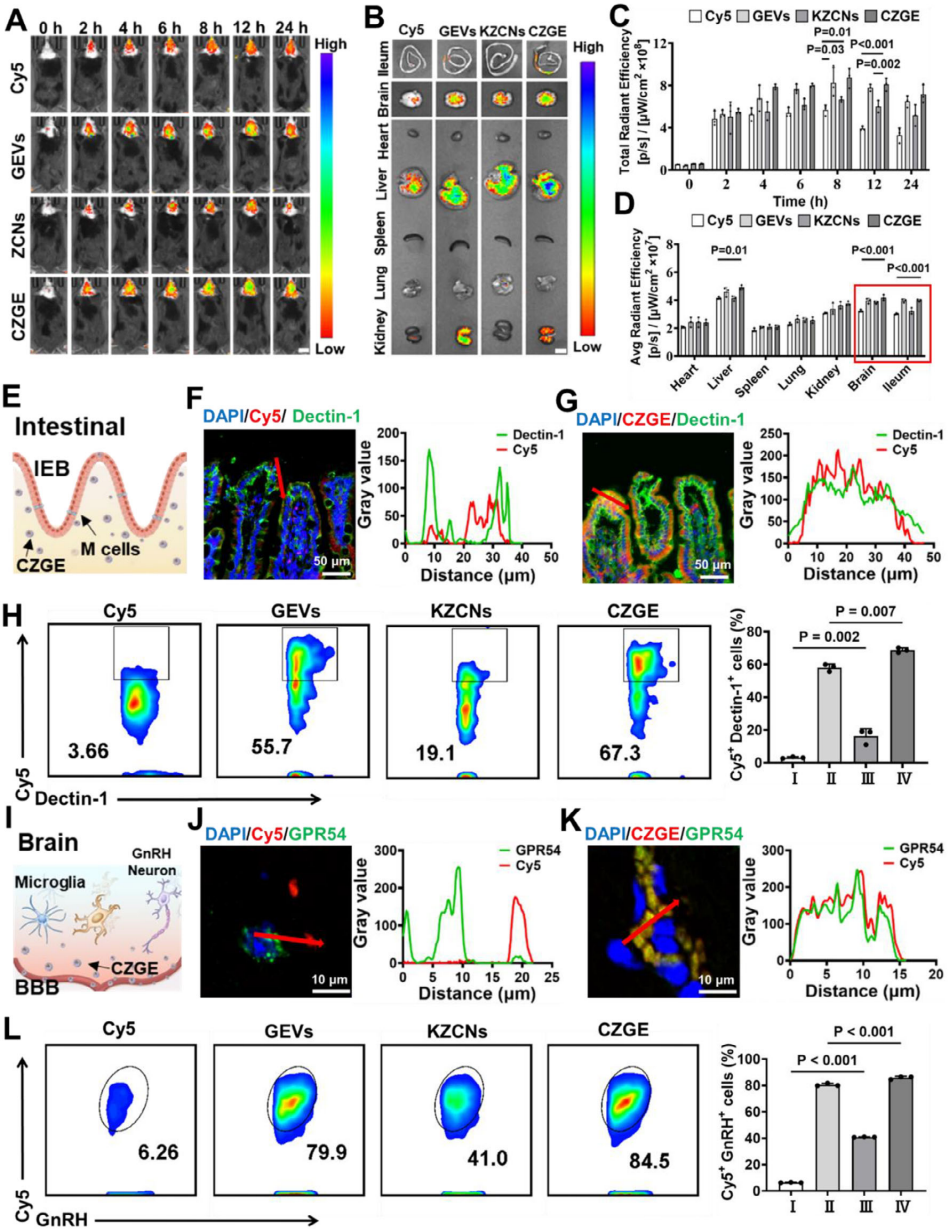
Evaluation of the target ability of CZGE in vivo. A,C) In vivo fluorescence imaging and the corresponding fluorescence quantification of WD mice administrated with Cy5‐labeled different samples at different time points, scar bar: 1 cm (*n* = 3). B,D) In vivo fluorescence imaging and the corresponding quantitative analysis of the ileum, brain and major organs from the normal and WD mice treated by different samples after 24 h of oral gavage (*n* = 3). E–G) Representative co‐localization images of different samples in the WD ileum after oral with different samples (blue: nucleus, green: Dectin‐1for M cells; red: free Cy5 or Cy5‐labled samples). H) Flow cytometry analysis and quantitative analysis of intestinal cells (Cy5^+^, Dectin‐1^+^) in the mice in each group (*n* = 3). J,K) Representative co‐localization images of different samples in the WD brain after oral with different samples (blue: nucleus, green: GPR54 for neuron; red: free Cy5 or Cy5‐labled samples). L) Flow cytometry analysis and quantitative analysis of hypothalamic cells (Cy5^+^, GnRH^+^) in the mice in each group (*n* = 3). (Samples for H and L, I: Cy5, II: GEVs, III: KZCNs, IV: CZGE). All statistical data are presented as mean ± standard deviation. Statistical analysis: two‐way ANOVA (C, D); one‐way ANOVA (H, L).

To further investigated the in vivo oral brain‐targeting capability of CZGE, the fluorescence distribution of various Cy5‐loaded samples in WD mice was monitored following oral gavage using an in vivo imaging system (IVIS). The fluorescence images revealed that the brain began to exhibit fluorescence signals 2 h post‐administration of different samples, with the most intense signals observed at 8 h (**Figure** **5**A,C). Then the brain and major tissues were collected for *ex vivo* fluorescence imaging and semi‐quantification after 24 h of post‐administered. It had been observed that CZGE can successfully traverse the intestinal barrier and exhibit significantly higher accumulation in the liver and brain compared to that in other groups (Figure 5B,D). These findings indicated that CZGE was capable of overcoming two critical biological barriers (IEB and BBB), thereby achieving targeted delivery to the brain following oral administration. Additionally, the oral doses of CZGE in various brain regions were calculated, which will be critical for the development of future oral brain‐targeting nanoparticle systems. The IVIS imaging results revealed the distribution of CZGE in the olfactory bulb (OB), cortex (CT), hypothalamus (HT), midbrain (MB), hippocampus (HC), and cerebellum (CB), with oral doses of 21.98%, 17.08%, 30.24%, 17.04%, 13.50%, and 9.22%, respectively. Notably, the highest concentration of CZGE was observed in the hypothalamus, which was the core region associated with reproductive dysfunction in WD and also the primarily distribution location of GnRH neurons (Figure S28, Supporting Information).

Previous in vitro studies had demonstrated that CZGE, rich in *β*‐glucan, could penetrate the IEB by targeting the Dectin‐1 receptor on the surface of intestinal M cells.^[^ ^44^, ^45^, ^46^ ^]^ Consequently, histological sections of the isolated tissue were stained to further elucidated the delivery pathway from the gut to the brain. The numerous CZGE particles (red color) were observed to effectively co‐localize with the Dectin‐1 receptor (green color) on intestinal M cells (Figure 5E–G). Subsequently, the targeting efficiency of CZGE in the intestinal was further investigated using in vivo flow cytometry. As shown in Figure 5H, the proportions of cells co‐positive for Cy5 and intestinal M cells in the ileum of mice treated with GEVs and CZGE were 58.1% and 68.7%, respectively. These values were 3.56 and 4.21 times higher than those in the KZCNs group. It was further demonstrated that CZGE can selectively target intestinal M cells and pass through IEBs, a process facilitated by the *β*‐glucan on their surface. Furthermore, the co‐localization of CZGE with GnRH neurons in the hypothalamus had demonstrated that the red fluorescence of the Kiss‐10 peptide effectively overlapped with the green fluorescence of GPR54, a receptor on the membrane surface of GnRH neurons (Figure 5I–K). Subsequently, the targeting efficiency of CZGE after penetrating the BBB was further verified in vivo. It was found that the number of cells co‐expressing Cy5 and GnRH in the hypothalamus of mice treated with GEVs and CZGE were 80.5% and 85.8%, respectively, which was 1.97 and 2.10 times higher than that of KZCNs (Figure 5L and Figure S29, Supporting Information). Collectively, these results provided strong evidence that the *β*‐glucan on the surface of CZGE could be utilize to sequentially cross the IEB and BBB, successfully delivering to the hypothalamus and targeting GnRH neurons with the Kiss‐10 peptide to enhance its accumulation in the WD hypothalamus, thereby achieving efficient oral brain targeting.

### 2.6 Assessment of the Therapeutic Mechanisms of CZGE in WD Mice

**Figure 6:**
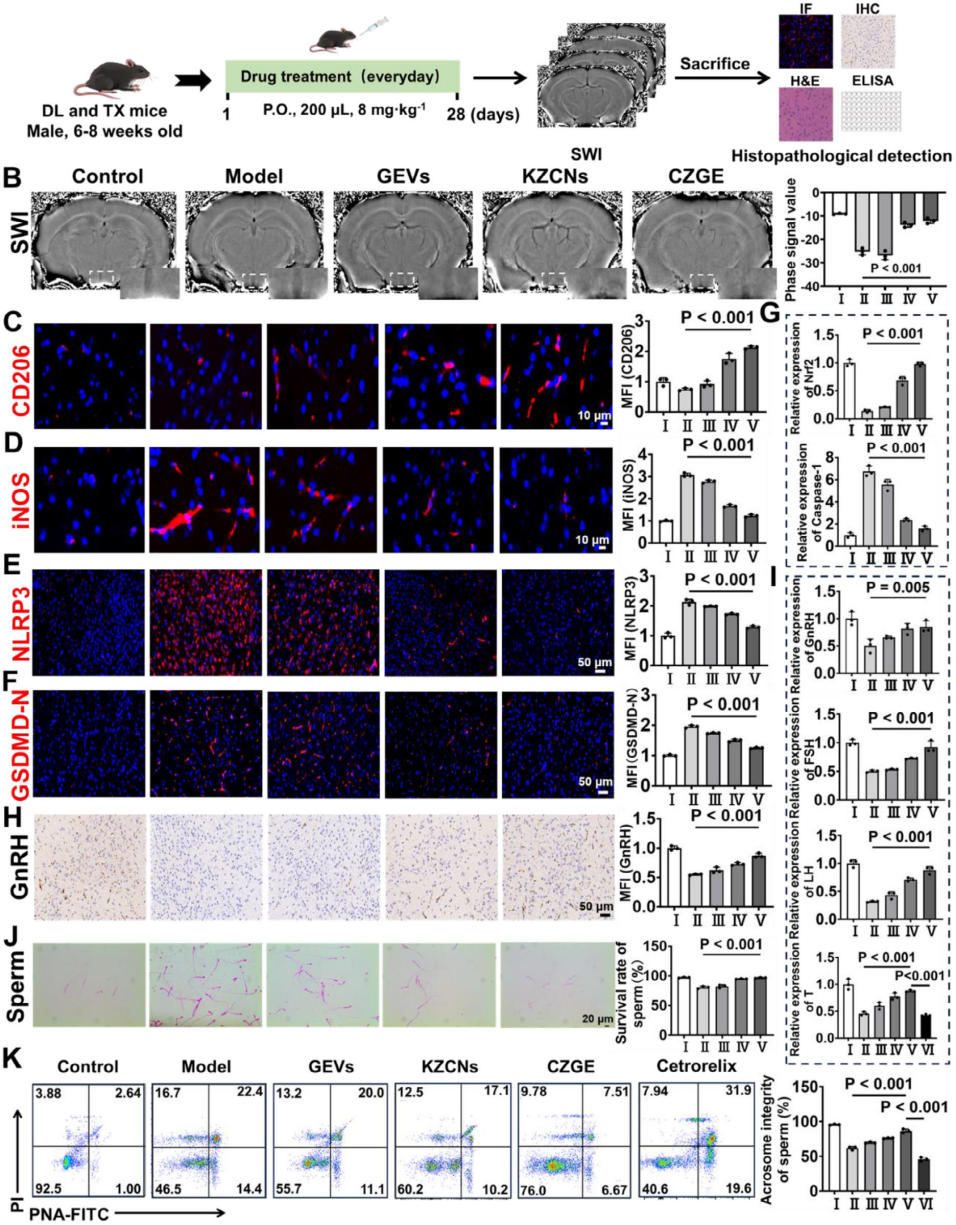
Assessment of the therapeutic mechanisms of CZGE in WD mice. A) The treatment schedule of the experiment. B) Representative SWI images and phase signal value analysis in the hypothalamus. C) Immunofluorescence staining and quantitative analysis of CD206 (red) in the hypothalamus of mice by different treatment (*n* = 3). D) Immunofluorescence staining and quantitative analysis of iNOS (red) in the hypothalamus of mice by different treatment (*n* = 3). E) Immunofluorescence staining and quantitative analysis of NLRP3 (red) in the hypothalamus of mice by different treatment (*n* = 3). F) Immunofluorescence staining and quantitative analysis of GSDMD‐N (red) in the hypothalamus of mice by different treatment (*n* = 3). G) Relative quantification of Nrf2, Caspase‐1 in the hypothalamus of mice by different treatments (*n* = 3). H) Immunohistochemical staining and quantitative analysis of GnRH in the hypothalamus of mice by different treatment (*n* = 3). I) Relative quantification of GnRH, FSH, LH and T in the serum of mice by different treatments (*n* = 3). J) The sperm survival rate for the mice after different treatments by an optical microscope (*n* = 3). K) The sperm plasma‐membrane and acrosome integrity for the mice after different treatments by flow cytometry (*n* = 3). (Samples for B‐K: I: Cy5, II: GEVs, III: KZCNs, IV: CZGE, VI: cetrorelix). All statistical data are presented as mean ± standard deviation. Statistical analysis: one‐way ANOVA.

As illustrated in **Figure** **6**A, the efficacy of CZGE in treating WD with reproductive dysfunction was further evaluated using TX mice, a recognized animal model for WD. Homozygous male toxic milk (TX) mice (6–8 weeks old) were randomly divided into model, GEVs, KZCNs, and CZGE groups, with homologous Dunn‐Lewis (DL) mice serving as the control group. For therapeutic intervention, oral gavage administration of 8 mg·kg^−1^ was performed daily for 28 days, with the control group receiving an equivalent dose of PBS. Post‐treatment evaluation of therapeutic efficacy was conducted using susceptibility‐weighted imaging (SWI) imaging and histopathological analysis.

First, SWI was used to dynamically assess the therapeutic effects of CZGE on the brains of WD mice (Figure 6B). It significantly low‐signal dark areas in the hypothalamic region of WD mice compared to that in the control group. Quantitative analysis showed that treatment with CZGE prevented the sharp decline of phase values in the hypothalamus of WD mice, restoring them to almost the same level as that of the mice in the control group. It suggested that secondary damage caused by copper deposition in the WD hypothalamus can be effectively mitigated by CZGE. As anticipated, CZGE treatment reduced copper levels in hypothalamic tissue of WD mice using inductively coupled plasma mass spectrometry (ICP‐MS) (Figure S30, Supporting Information). Besides, the expression levels of pro‐inflammatory cytokines (TNF‐α and IL‐6) were significantly reduced, whereas the levels of anti‐inflammatory cytokines (IL‐10 and IL‐4) were elevated in the CZGE‐treated group compared to that in the model group, which elucidated the reduction of copper levels to effectively mitigate the cascading neural effects by CZGE (Figure S31, Supporting Information). Furthermore, microglia play a crucial role in brain inflammation in WD. Among these, Iba‐1, serving as an important indicator of neuroinflammation, was significantly upregulated in the WD group but was significantly downregulated following CZCG treatment (Figure S32, Supporting Information). And the inflammatory microenvironment shifted from a pro‐inflammatory state (the expression of iNOS) to an anti‐inflammatory state (the expression of CD206) in the hypothalamus of WD mice following CZGE processing (Figure 6C,D; Figures S33 and S34, Supporting Information).

To explore the anti‐inflammatory mechanism in vivo, the expression levels of key pyroptosis‐related proteins in the hypothalamus of WD mice were examined. The expression levels of NLRP3 and GSDMD‐N were markedly elevated in WD mice compared to the control group, whereas these levels were significantly reduced in mice treated with CZGE (Figure 6E,F; Figures S35 and S36, Supporting Information). Additionally, the expression levels of Caspase‐1, IL‐18, and IL‐1*β* in the hypothalamus of the CZGE‐treated mice were notably decreased, while Nrf2 expression was significantly increased (Figure 6G and Figure S37, Supporting Information). This result corresponds to the ability of CZGE to effectively inhibit pyroptosis of hypothalamic neurons in vitro by upregulating Nrf2. Besides, the co‐expression of GnRH and NLRP3, as well as GnRH and PKC, in the hypothalamus of WD mice was further examined. It was found that in the WD model group, the expression of GnRH in the hypothalamus was significantly decreased, the expression of NLRP3 was significantly increased, and the expression of PKC was significantly decreased. Following treatment with CZGE, the expression of GnRH increased, the expression of NLRP3 decreased significantly, and the expression of PKC increased significantly (Figure S38, Supporting informing). These findings further confirm the hypothesis that copper deposition can affect the function of GnRH neurons by regulating the NLRP3‐PKC pathway. In addition, the zinc levels in hypothalamic tissue of the KZCNs and CZGE groups were found to be significantly higher than those in the model group, indicating that the nanomaterial successfully facilitated ion exchange and released Zn^2+^ in the brain within a high‐copper environment (Figure S39, Supporting Information). Urinary and fecal copper levels in WD mice were measured, and it was found that, compared with the model group, both urinary and fecal copper levels were significantly elevated in the KZCNs and CZGE groups, with the most pronounced increase observed in the CZGE group (Figure S40, Supporting Information). This further confirmed that the synergistic therapeutic effect of CZGE is achieved by inhibiting copper absorption and promoting copper excretion through the release of Zn^2+^, in addition to chelating intracellular copper.

The HPT axis plays a crucial role in regulating reproductive function. Specifically, the most important secretion GnRH by GnRH neurons can promote the production of secretions that promote sperm or testicular function (such as LH and FSH) by specifically binding to the specific protein (GnRHR I) of pituitary cells, thereby affecting reproductive function (Figure S41, Supporting Information). Therefore, the hormone secretion capability of the hypothalamus was initially evaluated using immunohistochemical staining. Compared to that in the control group, GnRH expression in the hypothalamus was significantly reduced in the model group but markedly increased in the CZGE‐treated group (Figure 6H). Subsequently, the causal relationship between copper deposition and GnRH neuronal injury was further investigated. The results demonstrated that, compared with that in the control group, red fluorescence (Cu^2+^) in the model group was markedly enhanced, whereas green fluorescence (GnRH) was significantly reduced. This suggested that copper deposition in the hypothalamic tissue of WD mice was substantially increased, leading to the inhibition of GnRH expression. However, following CZGE treatment, the red fluorescence intensity of Cu^2+^ in the hypothalamic neurons of WD mice decreased to a level comparable to that of the control group, while the green fluorescence intensity of GnRH was significantly elevated to 1.57 times that of the model group (Figure S42, Supporting Information). These findings indicated that excessive Cu^2+^ can markedly suppress the expression and secretion of GnRH, whereas CZGE can restore GnRH secretion by reducing Cu^2+^ levels. Additionally, the serum levels of GnRH, FSH, LH, and T in the mice of the model group were found to be significantly decreased compared with that in the control group. However, following treatment with CZGE, these levels were observed to have increased significantly in Figure 6I. Furthermore, the potential of CZGE to enhance testicular prognosis and function through the regulation of GnRH secretion and the utilization of the HPT axis was further explored using the GnRH secretion inhibitor (cetrorelix). It was observed that, compared to that in the CZGE group, the serum level of T in mice treated with cetrorelix was significantly reduced (Figure 6I). As the primary organ responsible for sperm production and testosterone secretion, the structure and function of the testis play a crucial role in reproductive function.^[^ ^47^, ^48^, ^49^ ^]^ Through hematoxylin and eosin (H&E) staining, structural changes in testicular tissue were observed, with damage to seminiferous tubules and necrosis of spermatogenic cells at all stages noted in the model group. In contrast, after CZGE treatment, spermatogenic cells were found to be more densely arranged. Meanwhile, spermatogenic tubules in the cetrorelix group exhibited varying degrees of damage and necrosis (Figure S43, Supporting Information). These findings suggested that enhancing GnRH secretion in the hypothalamus can influence T secretion and improve testicular morphology through the HPT axis. High‐quality sperm is essential for reproductive function.^[^ ^50^ ^]^ Sperm function was further assessed to confirm reproductive recovery. Observations of sperm smears revealed a significant increase in sperm survival in the CZGE group compared to the model group (Figure 6J). Additionally, flow cytometry analysis revealed that, compared with the model group, treatment with CZGE significantly enhanced the integrity of the plasma membrane and acrosome of mouse sperm. In contrast, the integrity of the plasma membrane and acrosome of mouse sperm in the cetrorelix group was significantly compromised (Figure 6K and Figure S44, Supporting Information). These results indicated that spermatogenesis was promoted and sperm quality improved in WD mice by CZGE.

Furthermore, to evaluate the long‐term efficacy of CZGE, the observation period was extended for 4 weeks after the completion of treatment, and changes in reproductive function were re‐evaluated. It was found that, compared to that in the model group, the level of T, sperm plasma membrane and acrosome integrity, and spermatogenic tubule structure of mice in the CZGE group were all improved to varying degrees. These findings indicated that CZGE has a beneficial long‐term therapeutic effect (Figure S45, Supporting Information). In conclusion, CZGE was found to reduce hypothalamic copper deposition, inhibit neuroinflammation, regulate HPT axis function, and enhance sperm quality, thereby maintaining reproductive function. Additionally, CZGE demonstrated a beneficial long‐term therapeutic effect.

Finally, in‐depth biocompatibility and safety analyses in vivo to understand the potential systemic toxicity of CZGE. Weight changes in WD mice were monitored over 28 days post‐treatment, revealing no significant differences among groups (Figure S46, Supporting Information). Blood routine analysis and H&E staining of brain and major organs (heart, liver, spleen, lung, kidney and intestinal) were also examined in each group. The results showed that no significant changes were observed in routine blood levels, no abnormal pathological changes were found in major organs or tissues, and a significant improvement was observed in the structure of hypothalamic tissue post‐treatment (Figure S47, Supporting Information and Table S1, Supporting Information). These findings demonstrated that CZGE treatment was biocompatible and non‐toxic. Taken together, the above experimental findings revealed that CZGE served as a highly efficient and safe gut‐to‐brain drug delivery system for the oral treatment of WD with reproductive dysfunction.

### 2.7 The Therapy Mechanisms by Transcriptomics Analyses

**Figure 7:**
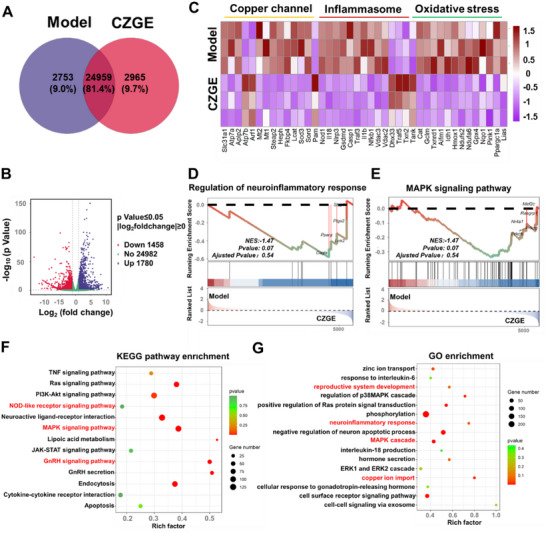
Transcriptional sequencing and biological analysis results. A) Venn diagram of differentially expressed genes in model and CZGE groups. B) Volcano plots showing the down‐regulated and up‐regulated genes between model and CZGE groups. C) Heatmap of expression levels of the refined gene classes (copper channel, inflammasome and oxidative stress) after the indicated treatments. D,E) Regulation of neuroinflammatory response and MAPK signaling pathway GSEA enrichment analysis. F) Dot plots of KEGG pathway enrichment analysis of the DEGs between the model and CZGE groups. G) GO enrichment analysis of DEGs in cellular biological process showing the most significantly enriched categories. *n*  =  3 biologically independent cell samples per group.

To further our understanding of the therapeutic process of CZGE, a comprehensive transcriptomic analysis of the hypothalamus was conducted in both the CZGE‐treated and model groups of mice, revealing potential therapeutic mechanisms at the genetic level. The Venn diagram illustrated that 24 959 genes were co‐expressed in the model and CZGE groups, while 2965 genes were exclusive in the CZGE group (**Figure** **7**A). Furthermore, volcano plot analysis of differentially expressed genes (DEGs) indicated that 1780 genes were up‐regulated and 1456 genes were down‐regulated in the CZCG treatment group (Figure 7B).

To delve deeper into the underlying mechanisms, heat maps were constructed to analyze gene expression patterns associated with copper channels, inflammasomes, and oxidative stress. The classification of DEGs revealed downregulation in genes associated with copper channels, inflammasomes, and oxidative stress, confirming that CZGE can inhibit pyroptosis and reduce inflammation at the genetic level (Figure 7C). Additionally, Gene Set Enrichment Analysis (GSEA) demonstrated that the neuroinflammatory response was actively regulated in the CZGE treatment group, with significant up‐regulation of the MAPK signaling pathway (Figure 7D,E). It aligned with previous studies and provided further evidence that CZGE plays a crucial role in GnRH secretion by inhibiting inflammation and modulating the MAPK‐PKC‐ERK pathway. The Kyoto Encyclopedia of Genes and Genomes (KEGG) enrichment analysis indicated significant enrichment of the NOD‐like receptor signaling pathway and the TNF signaling pathway among DEGs following CZGE treatment. Previous experimental studies had shown these pathways to be closely associated with the anti‐inflammatory therapeutic mechanisms of CZGE. Specifically, the NOD‐like receptor signaling pathway had been linked to pyroptosis, while ROS could induce systemic inflammatory responses through the activation of the TNF signaling pathway. Therefore, normalizing the expression levels of these genes could inhibit pyroptosis, thereby mitigating damage caused by copper deposition. Moreover, DEGs were significantly enriched in pathways regulating GnRH secretion, including the MAPK signaling pathway, GnRH signaling pathway, and Ras signaling pathway (Figure 7F).^[^ ^51^, ^52^, ^53^ ^]^ Genetic ontological (GO) enrichment analyses confirmed changes in copper ion import and activation of pathways involved in the regulation of neuroinflammatory responses, as well as alterations in biological functions related to reproductive system development before and after treatment (Figure 7G). The results of the transcriptomic analysis were also validated by qPCR revealed significant downregulation of hypothalamic inflammasome‐related gene (NLRP3), alongside significant upregulation of GnRH secretion‐regulating gene (MAPK14 and GnRH), consistent with the transcriptomic data (Figure S48, Supporting Information). Collectively, these findings suggested that hypothalamic neuronal pyroptosis was alleviated by CZGE through the reduction of hypothalamic copper deposition, thereby inhibiting inflammation, promoting GnRH secretion, and improving reproductive function to a certain extent.

## 3 Conclusion

In summary, an oral brain‐targeted drug delivery system via ion exchange strategy was developed and proposed for the treatment of WD with reproductive dysfunction. Specifically, the therapeutic system of CZGE, which incorporated the targeting functions of *β*‐glucan and Kiss‐10, can effectively surmount both the IEB and BBB, thereby accurately targeting the damaged neurons associated with WD lesions. Followed by internalization, the ion exchange replacement between excess Cu^2+^ and CZGE was rapidly occurred and released Zn‐Cur to form Cu‐Cur and the subsequent release of free Zn^2+^. The former exhibited anti‐inflammatory properties, effectively reducing in‐copper and neuroinflammation by promoting the Nrf2‐NLRP3 pathway, while Zn^2+^ blocks ex‐copper influx by activating ZnT1. Furthermore, it was demonstrated that oral administration of CZGE can significantly reduce WD‐related copper deposition, alleviate neuroinflammation, and markedly improve reproductive function. Given its high efficacy and favorable biosafety profile, CZGE was considered to hold significant potential for future clinical applications in treating WD with reproductive dysfunction.

## 4 Experimental Section

### Ethics Statement

All animal experiments were approved by the Laboratory Animal Management Committee of Institute of health and medicine, Hefei comprehensive national science center (Permit Number: IHM‐AP‐2024‐051). The use of human clinical indexes was approved by the Ethics Committee of the First Affiliated Hospital of Anhui University of Chinese Medicine (Permit Number: 2023AH‐56).

### Materials

All the Fmoc‐protected amino acids, Rink amide resins were purchased from Bide Pharmatech Co., Ltd. (Shanghai, China). 3,3′‐dioctadecyloxacarbocyanine perchlorate (DiO), DCFH‐DA, 5,5′,6,6‐Tetrachloro‐1,1′,3,3′‐tetraethyl‐imidacarbocyanineiodide (JC‐1) and cetrorelix were all purchased from Shanghai Beyotime Biotechnology Co., Ltd. (Shanghai, China). Zinquin ethyl ester was purchased from Maokang Biochemical Technology Co., Ltd. (Shanghai, China). Cu^2+^ fluorescence probe was purchased from Fount Biochemical Technology Co., Ltd. (Beijing, China). Curcumin, CuSO_4_·5H_2_O, Zinc acetate dihydrate, 1,2‐distearoyl‐sn‐glycero‐3‐phosphoethanolamine‐N‐ [methoxy (polyethylene glycol)]‐Cy5 (DSPE‐PEG‐Cy5), 1,1′‐dioctadecyl‐3,3,3′,3′‐tetramethylindocarbocyanine perchlorate (Dil) were all provided from Bide Pharmatech Co., Ltd. (Shanghai, China). Methyl‐*β*‐cyclodextrin (M*β*CD), amiloride hydrochloride were purchased from Aladdin Biochemical Technology Co., Ltd. (Shanghai, China). Chlorpromazine hydrochloride (CPZ) was purchased from Altascientific Co., Ltd. (Tianjin, China). • OH and SOD assay kit was purchased from Nanjing Success Biotechnology Research Institute Co., Ltd. (Nanjing, China). The Cell Counting Kit‐8 (CCK‐8) was purchased from Dojindo Laboratories (Kumamoto, Japan). NLRP3 inhibitor (MCC950) was purchased from Shandong Sparkred Biotechnology Co., LTD. Rabbit Anti‐Cleaved Caspase‐1, Rabbit Anti‐GSDMD, Rabbit Anti‐PKC, Rabbit Anti‐ERK1/2, Rabbit Anti‐p‐ERK1/2 were purchased from Jiangsu Kinko Biological Research Center Co., LTD. Rabbit Anti‐Nrf2, Mouse Anti‐NLRP3, Mouse Anti‐GnRH, Coralite488, FITC‐CD86 and PE‐CD206 were purchased from Wuhan Saneagle Biotechnology Co., LTD. DMEM/F12 (high glucose) and Pen‐Strep were purchased from Dalian MeilunBio Co., Ltd. Trypsin‐EDTA (0.25%) was purchased from Anhui KETU Biotechnology Co., Ltd. Mouse Tumor Necrosis Factor Alpha (TNF‐a), Mouse Interleukin‐4 (IL‐4), Mouse Interleukin‐10 (IL‐10) and Mouse Interleukin‐6 (IL‐6) ELISA Kits was purchased from Jianglai Industrial Co., Ltd. (Shanghai, China). Mouse Interleukin‐18 (IL‐18), Mouse Interleukin‐1*β* (IL‐1*β*), Mouse NLRP3 and Mouse PKC ELISA Kits was purchased from Meimian Industrial Co., Ltd. (Jiangsu, China).

### Instrumentation

The exosomes are obtained by ultra‐high centrifuge (OptimaXPN‐100, Beckman, USA). The Zeta potential was recorded by Zetasizer (ZEN3690, Malvern, USA). The Fluorescence resonance energy transfer (FRET) was detected by Fluorescence spectrophotometer (F4600, Japan). The ^1^H‐NMR spectrum was recorded on the Bruker ADVANCE III 600 MHz spectrometer. Transmission electron microscopy (TEM) images was obtained using Hitachi HT7800 (80 kV). The particle concentration and particle size of GEVs, KZCNs, and CZGE were detected by nanoparticle tracking analysis (NTA, Malvern, UK). All fluorescence images of cells were captured by CLSM (Zeiss LSM900, Germany) and Inverted fluorescence microscope (LEICA DMI8, Germany). Cell viability was detected by Microplate reader (SpectraMax i3X, USA). The clearance of intracellular ROS, and dysfunction of mitochondria were assessed using flow cytometry. (BD FACS Celesta, BD, USA). Biological distribution in vivo and in vitro was performed on the IVIS imaging system (PerkinElmer). In vivo magnetic resonance imaging of mice was performed on a 9.4 T MRI scanner (uMR 9.4T, Shanghai, China).

### Cell Lines and Animals

GT1‐7 cell lines were purchased from Meilun Biotechnology Co., LTD. (Dalian, China). Caco‐2 and bEnd.3 cell lines were purchased from Shanghai Xinyu Biotechnology Co., LTD. (Shanghai, China). The complete medium was prepared by adding 10% FBS and 1% Pen/Strep/Amp solution to basic medium DMEM/F12 or DMEM, and cultured in a 5% CO_2_ incubator at 37 °C. TX mice (C3He‐ATP7B^tx‐j^) carrying an ATP7B genemutation were purchased from the Jackson Laboratory Animal Center (Maine, USA).

### Isolation and Purification of GEVs

GEVs were isolated from ganoderma using differential ultracentrifugation. 50 g of dried ganoderma were soaked in 200 mL of ice‐cold phosphate‐buffered saline (PBS, 10 mM, pH 7.4) and processed in a blender to obtain the juice. The juice underwent centrifugation at 500 g for 10 min, 2000 g for 20 min and 10 000 g for 30 min to remove large ganoderma fibers. The supernatant was then subjected to further ultracentrifugation at 150 000 g for 2 h, followed by resuspension of the pellet in PBS. The GEVs was stored at −80 °C for future use, and the particle size and zeta potential of GEVs were measured by dynamic light scattering (DLS) and TEM.

### Preparation of Zn‐Cur and Cu‐Cur

The Zn‐Cur chelate was synthesized by mixing zinc acetate dihydrate with methanolic solution of curcumin at a molar ratio of 1:1. Specifically, 50 mL of methanolic solution of curcumin (2 mM) was prepared and then heated to 60 °C for 0.5 h. Concurrently, 2 mM of zinc acetate dehydrate was dispersed in 100 mL of methanol by magnetic stirring for 0.5 h. The prepared solution was then added to the curcumin solution under magnetic stirring. The product was subsequently filtered, rinsed three times with cold methanol, and freeze‐dried overnight for further use. The Cu‐Cur chelate was prepared similarly, substituting CuSO_4_·5H_2_O for zinc acetate dihydrate. It was subsequently characterized using ^1^H‐NMR spectrum, ultraviolet‐visible (UV–vis) absorption spectrum, and FTIR spectra.

### Preparation of ZCNs, KZCNs, and CZGE

ZCNs were prepared by a thin film hydration method according to previously reports. In brief, DSPE‐PEG_2000_‐Mal (2 mg) was dissolved in 4.5 mL of acetonitrile, while curcumin (1 mg) was dissolved in 4.5 mL of the mixture solvent of ethanol and methanol (1:2, v: v). The two solutions were combined in a round flask, and the organic solvent was removed by rotary evaporation to obtain a thin dried film. The dried was then hydrated with 20 mL of PBS at 60 °C for 30 min. After centrifugation at 10 000 rpm for 3 min to remove free curcumin to obtain ZCNs. Subsequently, the targeted peptide of Kiss‐10 was prepared by standard Fmoc solid‐phase peptide synthesis (SPPS) method, and 0.62 mg of Kiss‐10 was further added to the above ZCNs solution (10 mL), and the mixture was incubated under N_2_ protection for 12 h to obtain KZCNs. To investigate if Kiss‐10 were successfully grafted onto ZCNs surface, Dil‐labeled ZCNs were combined with FITC‐labeled Kiss‐10 according to the described procedure. The resulting mixtures were imaged using the inverted fluorescence microscope, and the fluorescence intensity was measured at 490 nm excitation wavelength and 520 nm emission wavelength using fluorescence spectroscopy. The same number of KZCNs and GEVs, quantified and extruded through 0.2 µm polycarbonate membrane filter 10 times and further merged for 12 h to obtain uniform CZGE. The particle size and zeta potential were characterized by DLS and TEM. The particle concentration and particle size of GEVs, KZCNs, and CZGE were detected by NTA.

### Vesicle Fusion Studies

GEVs and KZCNs were labeled with 20 µM DiO and Dil, respectively. The labeled GEVs and KZCNs were then incubated at room temperature for 12 h and extruded through a 0.2 µm polycarbonate membrane at a 1:1 ratio, followed by a further incubation for 12 h. Finally, the mixture was imaged using an inverted fluorescence microscope.

FRET assay was used to verify membrane fusion to check whether GEVs were incorporated into KZCNs. GEVs were labeled with Dil (excitation/emission = 549/565 nm) and DiD (excitation/emission = 644/663 nm). KZCNs were then added to the dye‐labeled GEVs at a 1:1 ratio for fusion. The fluorescence spectrum of KZCNs were measured within the 550 to 750 nm range, using an excitation wavelength of 525 nm.

### Antioxidation Performance and Mechanism of Cur, Zn‐Cur, and Cu‐Cur

The inhibition rates of •OH and O_2_^•‐^ for Cur, Zn‐Cur and Cu‐Cur at concentrations ranging from 20 to 120 µg mL^−1^ were determined by •OH and SOD detection kit. In addition, the ESR spectroscopy of Cur, Zn‐Cur and Cu‐Cur was performed using DMPO/BMPO as the spin trapping reagent. For O_2_^•‐^ detection assay, 1 mM of xanthine, 0.2 unit mL^−1^ of XO, 5 mM of BMPO and Cur, Zn‐Cur or Cu‐Cur (120 µg mL^−1^) were added into the PBS solutions. Subsequently, an aliquot of the solution (100 µL) was transferred into the quartz tube for ESR measurements. For •OH detection assay, 2 mM of H_2_O_2_, 10 µM of FeSO_4_, 1.5 of mM DMPO, and Cur, Zn‐Cur or Cu‐Cur (120 µg mL^−1^) were added into the PBS solutions.

### Evaluation of the Stability of GEVs, KZCNs, and CZGE

GEVs, KZCNs and CZGE were stored at 37 °C in 10% FBS to mimic blood conditions. Their size distribution was examined by DLS from 1 day to 7 days at a set time.

### Cell Viability Assay

About 1 × 10^4^ cells mL^−1^ GT1‐7 cells were inoculated in 96‐well plates and cultured for 24 h. In the stimulation group, CuSO_4_ with a final concentration of 300 µM was added and cultured at 37 °C for 24 h. Different concentrations of CZGE were added. After incubation for 24 h, CCK‐8 assay was utilized to evaluate the viability of GT1‐7 cells after different treatments. In addition, the experimental procedures for cell viability in different samples and at different times were consistent with the above.

### Experiment of a Simulated IEB and BBB—In the IEB Mimetic Model

The Caco‐2 cells and RAW264.7 cells were mixed in a 9:1 ratio were seeded with a density of 1 × 10^5^ cells mL^−1^ in the upper chambers (12 well, pore size: 0.4 µm). After a 7‐day incubation period, the IEB was successfully formed. RAW264.7 cells were then seeded in the lower chambers. The IEB was then stimulated with CuSO_4_​ (300 µM) for 24 h. Subsequently, different Cy5‐labeled samples (10^6^ particle mL^−1^) were introduced into the upper chamber and incubated for an additional 8 h. In the Dectin‐1 inhibitor group, Laminarin (1 mg mL^−1^) was pre‐incubated for 3 h prior to the addition of CZGE labeled with Cy5 for further incubation.

### In the BBB Mimetic Model

The bEnd.3 cells were seeded with a density of 5 × 10^4^ cells mL^−1^ in the upper chambers (12 well, pore size: 0.4 µm) for 10 days to form the blood brain barrier layer. GT1‐7 cells were then seeded in the lower chambers, treated with 300 µM CuSO_4_ for 24 h. Following this, different Cy5‐labeled samples (10^6^ particle mL^−1^) were added to upper chamber and incubated for 24 h. After discarding the culture medium, the cells in the lower and upper chambers were collected and were stained with DAPI (5 µg mL^−1^) for 10 min, respectively. The stained GT1‐7 cells were recorded by CLSM and IVIS system.

Penetration rate (%) = Fluorescence intensity of Cy5 from the lower chambers /Fluorescence intensity of Cy5 from the lower chambers and the upper chamber × 100%.

### Cellular Uptake

GT1‐7 cells with a density of 5 × 10^4^ cells mL−^1^ were inoculated in confocal dishes. A final concentration of 300 µM of CuSO_4_ was stimulated for 24 h. Different samples were added to confocal dishes at different times (1, 2, and 4 h) for co‐culture. Then, the cells were cleaned with PBS and stained with DiO (10 µM) and DAPI (5 µg mL^−1^) for 15 min. After washing with PBS, fluorescence images of GT1‐7 cells were detected by CLSM. To quantitatively measure the level of cell uptake, GT1‐7 cells were grafted as described above, collected and cleaned for flow cytometry analysis.

### Endocytosis Mechanism Assay

To explore the endocytic pathways in GT1‐7 cells, various endocytic inhibitors were introduced. GT1‐7 cells (5 × 10^4^ cells mL^−1^) were seeded in 24‐well plates. For stimulation, CuSO_4_ was administered to the designated group at a final concentration of 300 µM and incubated at 37 °C for 24 h. To assess the impact of endocytosis inhibitors, specific inhibitors were added: chlorpromazine (CPZ, 10 µg mL^−1^), amiloride (1 mM) and methyl‐*β*‐cyclodextrin (M*β*CD, 500 µM) for caveolin‐mediated endocytosis. These inhibitors were incubated for 4 h at 4 °C. Following this, DiO‐labeled samples (10^6^ particle mL^−1^) were introduced and incubated for an additional 4 h. Subsequently, the cells were collected, and the DiO fluorescence signal was measured using flow cytometry.

### Analysis of Intracellular Zn^2+^ and Cu^2+^ Level

GT1‐7 cells were seeded into culture dishes at a density of 5 × 10^4^ cells mL^−1^ and cultured for 24 h. Subsequently, the cells were stimulated with a 300 µM CuSO_4_ solution and incubated at 37 °C for 24 h. Various samples, at a concentration of 10^6^ particle mL^−1^, were then added and incubated for an additional 24 h. Then, 200µL of zinquin ethyl ester (20 µM) or 200 µL of Cu^2+^ fluorescence probe (1 µM) was added in the dark at 37 °C for 30 min. After washing with PBS three times, the cells were collected to observe and detect using fluorescence microscopy and flow cytometry analysis.

The GT1‐7 cells were plated in the same experimental manner. The cells were incubated with CuSO_4_ (300 µM) for 24 h, followed by the addition of the CZGE* (10^6^ particle mL^−1^) for 4 h, and then CuSO_4_ (300 µM) was added for stimulation. A Cu^2+^ fluorescent probe (1 µM) was used for incubation at 37 °C in the dark for 30 min and subsequent flow cytometry detection.

The GT1‐7 cells were plated in the same experimental manner and incubated with CuSO_4_ (300 µM) for 24 h. Si‐ZnT1 (Huzhou Hippobio Co., LTD.) was then transfected, followed by incubation with CZGE* (10^6^ particle mL^−1^) for 4 h, after which CuSO_4_ was added for stimulation. Subsequently, 200 µL of a Cu^2+^ fluorescent probe (1 µM) was added to the cells, which were then incubated at 37 °C in the dark for 30 min, followed by flow cytometry detection. Following the same experimental procedure, CZGE** (10^6^ particle mL^−1^) was added for 4 h, and a Cu^2+^ fluorescent probe was used for incubation and subsequent flow cytometry detection.

### Analysis of Intracellular ROS Level

GT1‐7 cells, at a density of 5 × 10^4^ cells mL^−1^, were inoculated into confocal dishes and cultured overnight. Following induction with 300 µM CuSO_4​_ for 24 h, various samples were introduced and incubated for an additional 24 h. The cells were then washed three times with PBS and stained with 2 µM DCFH‐DA for 30 min. Subsequently, the cells were observed under an inverted fluorescence microscope. and prepared for flow cytometry analysis.

### Determination of Mitochondrial Membrane Potential

The mitochondrial membrane potential was evaluated using a JC‐1 fluorescence probe assay kit. GT1‐7 cells were seeded into culture dishes at a density of 5 × 10^4^ cells mL^−1^ per well and cultured for 24 h. Subsequently, the groups designated for stimulation were treated with CuSO_4​_ at a final concentration of 300 µM and incubated at 37 °C for 24 h. After that, different samples (10^6^ particle mL^−1^) were added to the culture dish and incubated for 24 h. The cells were then washed and incubated with JC‐1 probe (5 µg mL^−1^) for 30 min. Finally, the cells were washed three times with PBS, and the fluorescence signal was analyzed using flow cytometry.

### Evaluation of BV2 Cell Polarization

The polarization of M1 microglia was evaluated by immunofluorescence staining (CD86 representing M1 phenotype and CD206 representing M2 phenotype). A density of 5 × 10^4^ BV2 cells were inoculated in a 6‐well plate. After CuSO_4_ (300 µM) induction for 24 h, media containing GEVs, KZCNs and CZGE (10^6^ particle mL^−1^) were added, respectively. After 24 h, the cells were washed with PBS and incubated with fixation/osmotic buffer (BD Biosciences, 554 722) for 20 min, and incubated with perm/wash buffer (BD Biosciences, 554 723) for 15 min. Subsequently, BV2 cells were collected and labeled with antibodies FITC‐CD86 (PTG, 65 068) and PE‐CD206 (PTG, 141 705). After washing with flow cytometry staining buffer, the suspended cells were detected by flow cytometry immediately.

### Enzyme‐Linked Immunosorbent Assay

GT1‐7 cells were plate and treated according to the above method, and cells treated with different samples were collected. Among them, the NLRP3 inhibitor group was treated with MCC950 (10 µM) for 24 h. The cell suspension was diluted with PBS, and the cell was broken by ultrasonic wave to destroy the cell and release the intracellular components, and the supernatant was carefully collected after centrifugation at 2–8 °C for 20 min (2000–3000 r min^−1^). The levels of IL‐1*β*, IL‐18, IL‐6, TNF‐α, IL‐4, IL‐10, NLRP3 and PKC were then measured by ELISA kit.

### Western Blotting Experiment

GT1‐7 cells with a density of 5 × 10^5^ were inoculated in 6‐well plates overnight and induced by CuSO_4_ (300 µM) for 24 h. PBS was washed twice, and 1mL medium containing PBS, GEVs, KZCNs, CZGE (10^6^ particle mL^−1^) was treated for 24 h, respectively. After collection, the medium was suspended with 100 µL lysis buffer, store on ice for a few minutes to use, and ultrasonic cracking, 10 000 rpm centrifuge for 20 min to take supernatant for use. After the protein concentration was determined with the BCA protein detection kit (Jiangsu KeyGEN BioTECH Corp., Ltd, KGA902), appropriate samples were absorbed by SDS‐PAGE electrophoresis, and then transferred to PVDF membrane. Closed with skim milk powder and Rabbit Anti‐ZnT1 (1:2000; Bioss, bs‐6440R) was incubated at 4 °C overnight, cleaned by TBST, and incubated at room temperature for 1 h (1:2000). Finally, the protein signal was detected by ECL reagent and analyzed by GAPDH normalized Gel‐Pro32.

### Confocal Microscopy Experiments

Real‐time confocal fluorescence imaging of Zn^2+^: GT1‐7 cells were seeded in a confocal dish at a density of 5 × 10^4^ cells mL^−1^ and stimulated with 300 µM CuSO_4_ for 24 h. Subsequently, 200 µL of Zinquin ethyl ester (20 µM) was added, and the cells were incubated at 37 °C for 30 min in the dark. After recording was started, CZGE (10^6^ particle mL^−1^) was introduced into the dish, and fluorescence was recorded over a period of 10 min. The fluorescence intensity of Zn^2+^ was quantified on a per‐minute basis.

Real‐time confocal fluorescence imaging of Cu^2+^: GT1‐7 cells were seeded in a confocal dish with a density of 5 × 10^4^ cells mL^−1^, supplemented with 300 µM CuSO4 for 24h, rinsed with PBS, CZGE (10^6^ particle mL^−1^) was added to the dish and incubated for 4 h, and then 200 µL Cu^2+^ fluorescence probe (1 µM) was added, and the cells were incubated at 37 °C for 30 min in the dark. After recording was started, CuSO4 (300 µM) was added to the dish, the fluorescence was recorded for 10 min, and the fluorescence intensity of Cu^2+^ was quantified per minute. In addition, GT1‐7 cells without CZGE (10^6^ particle mL^−1^) were incubated with the same method as above to observe the changes of Cu^2+^ fluorescence intensity within 10 min.

### In Vitro Co‐Culture Experiment Modeling the Hypothalamic‐Pituitary‐Testicular Axis

GT1‐7 cells were inoculated at a density of 5 × 10^4^ cells mL^−1^ in the upper chamber (12 well, pore size: 0.4 µm), and the primary pituitary cells of TX mice were extracted and inoculated in the lower chamber. After treatment with 300 µM CuSO_4_ for 24 h, different samples (10^6^ particle mL^−1^) were added to the upper chamber and incubated for 24 h. The supernatant of the upper and lower chambers was collected and centrifuged, and the hormone levels in the supernatant were detected according to the instructions of GnRH, FSH and LH ELISA kits. In addition, TM3 cells were inoculated into 12‐well plates at a density of 5 × 10^4^ cells mL^−1^, and cultured with the collected supernatant in the lower chamber for 24 h. After the supernatant was collected and centrifuged, T levels in the supernatant were detected according to the instructions of the T ELISA kit.

### Animals and Administration

Male homozygous TX mice aged 6 to 8 weeks were randomly allocated into four experimental groups: model group, GEVs group, KZCNs group, and CZGE group. Homologous DL mice served as the control group (n = 8 per group). Mice in the experimental groups received daily administrations of respective samples (200 µL, 8 mg·kg^−1^), whereas those in the control group received an equivalent volume of PBS for a total duration of 28 days. Among that, the GnRH inhibitor group received daily intraperitoneal injections of cetrorelix (5 µg kg^−1^) during the last 7 days of treatment with CZGE.

### In Vivo Bio‐Distribution Assay and Pharmacokinetic Study

To evaluate the in vivo distribution of nanoparticles, WD mice were administered 200 µL of free Cy5 and various Cy5‐labeled samples (8 mg·kg^−1^) via oral gavage. Subsequently, imaging was conducted using the IVIS system at 2, 4, 6, 8, 12, and 24 h post‐administration. For *ex vivo* imaging, the mice were sacrificed after in vivo imaging, and brains, iluem, hearts, livers, spleens, lungs, and kidneys were collected. The brain tissue of mice was dissected into the olfactory bulb, cortex, hypothalamus, midbrain, hippocampus, and cerebellum, and subsequently homogenized. The Cy5 fluorescence signal in each organ was visualized using IVIS.

To examine the pharmacokinetic properties of the nanoparticles, WD mice were given 200 µL of free Cy5 and Cy5‐labeled CZGE (8 mg·kg^−1^) by oral gavage. Blood samples (1 mL) were collected from the mice at 0.5, 2, 4, 6, 8, 12, and 24 h post‐administration, centrifuged at 4 °C for 10 min (4000 rpm), and the fluorescence intensity of Cy5 in the supernatant was measured using a fluorescence spectrometer.

### Stability of CZGE under GI Conditions

To assess the stability of various samples under gastrointestinal conditions, in vitro studies were conducted. Samples were incubated in simulated gastric fluid (SGF, pH 1.8, prepared with 10% hydrochloric acid) and simulated intestinal fluid (SIF, pH 7.4, phosphate‐buffered saline) at 37 °C. Specifically, samples were exposed to SGF for 2 h followed by SIF for 4 h. Aliquots were collected at 0, 1, 2, and 4 h, and the drug content was subsequently analyzed.

### Flow Cytometry Analysis In Vivo

To evaluate the in vivo targeting efficiency of nanoparticles after penetrating the IEB, WD mice were randomly assigned to different groups administered 200 µL of free Cy5 and various Cy5‐labeled samples (8 mg·kg⁻¹) via intragastric gavage. Then, the mice were euthanized, and the ileum tissue was promptly excised and finely minced after 8 h. The tissue was then completely immersed in 2 mL of digestion solution (RPMI‐1640 containing 1 mg mL^−1^ of collagenase IV). The above mixture was digested at 37 °C for 30 min and then added to complete medium to terminate the digestion reaction. A 70 µM cell filter was used to further remove inadequately digested tissue and cell masses, yielding an ileal tissue suspension. Following fixation and permeabilization, the cells were incubated with rabbit anti‐Dectin‐1 (1:300, PTG, 22809‐1‐AP) overnight at 4 °C. Subsequently, CoraLite488 (1:500, PTG, SA00013‐2) was applied for 1 h in the dark. Flow cytometry was employed for analysis.

To evaluate the in vivo targeting efficiency of nanoparticles after penetrating the BBB, WD mice were administered 200 µL of free Cy5 and various Cy5‐labeled samples (8 mg·kg⁻¹) via intragastric gavage. After 24 h, the mice were sacrificed, and the hypothalamic tissues from each group were immediately isolated and fully homogenized in PBS at 4 °C. Hypothalamic cells were subsequently extracted through filtration and centrifugation. Following fixation and permeabilization, the cells were incubated with rabbit anti‐GnRH (1:200, PTG, 26950‐1‐AP) overnight at 4 °C. Subsequently, CoraLite488 (1:500, PTG, SA00013‐2) was applied for 1 h in the dark. Flow cytometry was employed for analysis.

### SWI Imaging

In vivo magnetic resonance imaging was performed on a 9.4 T MRI scanner (uMR 9.4T, Shanghai, China). During the scanning, the mice were anesthetized by inhaling isoflurane, and the mice's heart rate and respiration were monitored in real time. For analysis, the hypothalamic arcuate nucleus was manually defined as a region of interest (ROI) in imaging slices of each mouse. The phase value was measured on the phase diagram.

### Immunofluorescence Staining and Measurement of Brain Cytokines

After the treatment, the major organs (heart, liver, spleen, lungs, kidneys, brain, iluem, testicle) of mice in each experimental group were collected and fixed in 4% paraformaldehyde. Then, the brain was paraffin embedded to cut into sections of 3 µm in thickness. Immunofluorescence staining was performed to detect Iba‐1, CD206, iNOS, GSDMD‐N and NLRP3 in the hypothalamus region of the brain. Additionally, immunofluorescence staining was employed to detect the co‐expression of Cu^2+^ with GnRH, GnRH with NLRP3, and GnRH with PKC in the hypothalamus region of the brain. GnRH expression in hypothalamus was detected by immunohistochemical staining. Subsequently, the organs were embedded in paraffin and 5 µm sections were prepared for hematoxylin and eosin (H&E) staining. Furthermore, the levels of cytokines (Nrf2, Caspase‐1, IL‐1*β*, IL‐18, TNF‐α, IL‐10, IL‐6, IL‐4) in the brain hypothalamus of each group were quantified using specific assay kits.

### Measurement of Sperm Survival Rate

A 4 µL aliquot of eosin Y stain was applied to a glass slide and combined with an equal volume (4 µL) of sperm suspension. After 3 min, the slide was covered with a coverslip and examined under an optical microscope. Normal sperm appeared unstained, whereas dead sperm exhibited pink staining due to cell membrane rupture. Then, 200 sperms were randomly counted, and the ratio of unstained live sperm to total sperm was calculated.

### Measurements of Sperm Plasma Membrane and Acrosome Integrity

A 100 µL volume of sperm suspension was introduced into a 1.5 mL centrifuge tube and sequentially stained with isothiocyanate‐fluoresceinated peanut agglutinin (PNA‐FITC) and propidium iodide (PI). The integrity of the sperm plasma membrane and acrosome was assessed using flow cytometry. PI‐ indicated the integrity of the sperm plasma membrane, while PNA‐ indicated the integrity of the sperm acrosome.

### Quantitative Real‐Time PCR

Total RNA was extracted from hypothalamus tissue using TRIzol reagent (15 596 018, Life Technologies, New York, USA) and processed with chloroform, isopropanol, and 75% ethanol following homogenization. The integrity and purity of the RNA were assessed using a microspectrophotometer. RNA was reverse‐transcribed into cDNA and subsequently amplified by PCR. The amplification conditions were as follows: pre‐denaturation at 95 °C for 1 min, denaturation at 95 °C for 20 s, annealing and extension at 60 °C for 1 min, for a total of 40 cycles. The relative expression of the target genes was calculated using the 2^‐ΔΔCt^ method. Primer sequences are listed in Table S2, Supporting Information.

### Statistical Analysis

All experiments were conducted a minimum of three times. Data are presented as means ± standard errors. Statistical significance was assessed using Student's *t*‐test for pairwise comparisons and one‐way or two‐way ANOVA with Tukey's post hoc test for multiple comparisons.

## Conflict of Interest

The authors declare no conflict of interest.

## Supporting information



Supporting Information

## Data Availability

The main data generated in this study are provided in the article and Supplementary Information. Additional data are available from the corresponding author on request.
