# Supplementary material for: Oral Engineered Extracellular Vesicles Based on Ion Exchange Strategy for Multipronged Management of Wilson's Disease Complicated with Reproductive Dysfunction Therapy
Source: Adv Sci (Weinh). 2025 Jul 17;12(38):e01689. doi: 10.1002/advs.202501689 (PMC12520522; doi:10.1002/advs.202501689)
Supplement: Supplementary file 1 — Supporting Information [file ADVS-12-e01689-s001.docx]

Supplementary Information

**Oral engineered extracellular vesicles based on ion exchange** **strategy for** **multipronged management** **of** **Wilson's disease complicated with reproductive dysfunction** **therapy**

*Tingting Wang^†^, Wengui Lu^†^, Zhifei Cheng, Luyao Wang, Zhenzhen Jiang, Yike Yue, Pengyu Jiang, Zehua Xia, Lei He, Fengying Wang, Limin Wu*, Qi Wang* and Hui Han**

T. Wang, L. Wang, Z. Jiang, Y. Yue, P. Jiang, Z. Xia, L. He, F. Wang, H. Han

Department of Neurology, The First Affiliated Hospital of Anhui University of Chinese Medicine, Hefei, Anhui 230031, China.

E-mail: hanhuidoctor2022@163.com

W. Lu, Z. Cheng, Q. Wang

Anhui Province Key Laboratory of Pharmaceutical Preparation Technology and Application, School of Pharmacy, Anhui University of Chinese Medicine, Hefei, Anhui 230012, China.

E-mail: wangqi-njnu@ahtcm.edu.cn

L. Wu

Center for Reproduction and Genetics, The First Affiliated Hospital of USTC, Division of Life Sciences and Medicine, University of Science and Technology of China, Hefei, Anhui, 230001, China.

E-mail: wlm@ustc.edu.cn

^†^These authors contributed equally to this work and should be considered co-first authors.

**Supplementary Figures and Tables**

**
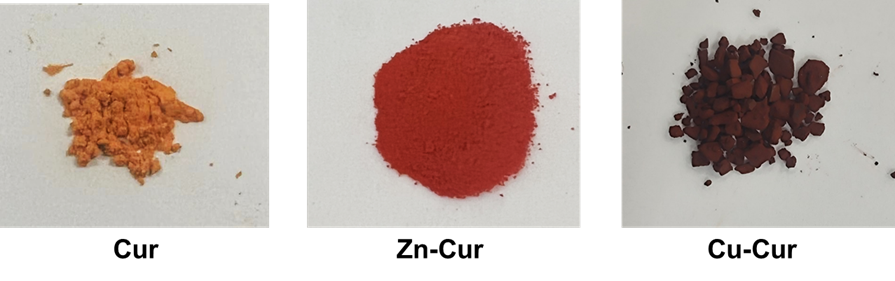
**

**Figure S1.** Digital photos of the powder of Cur, Zn-Cur, and Cu-Cur.


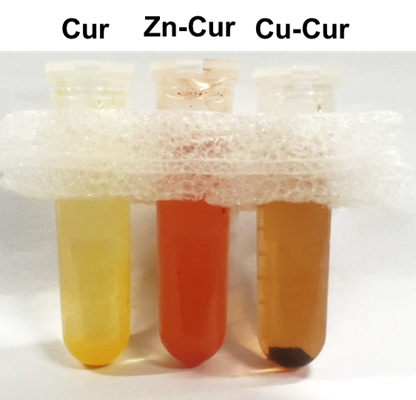


**Figure S2.** Digital photos of Cur, Zn-Cur, and Cu-Cur dispersed in water.


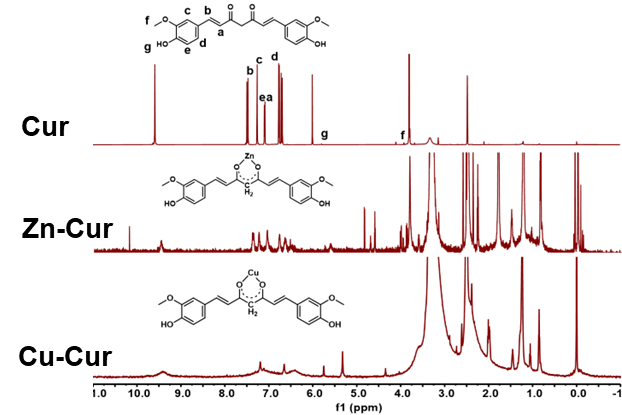


**Figure S3.** Solution ^1^H NMR spectra of Cur, Zn-Cur and Cu-Cur in DMSO.

**
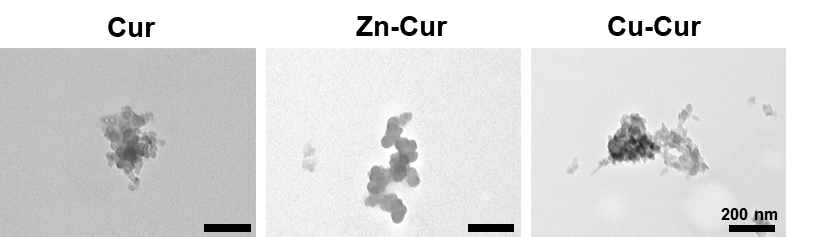
**

**Figure S4**. TEM images of Cur, Zn-Cur, and Cu-Cur.


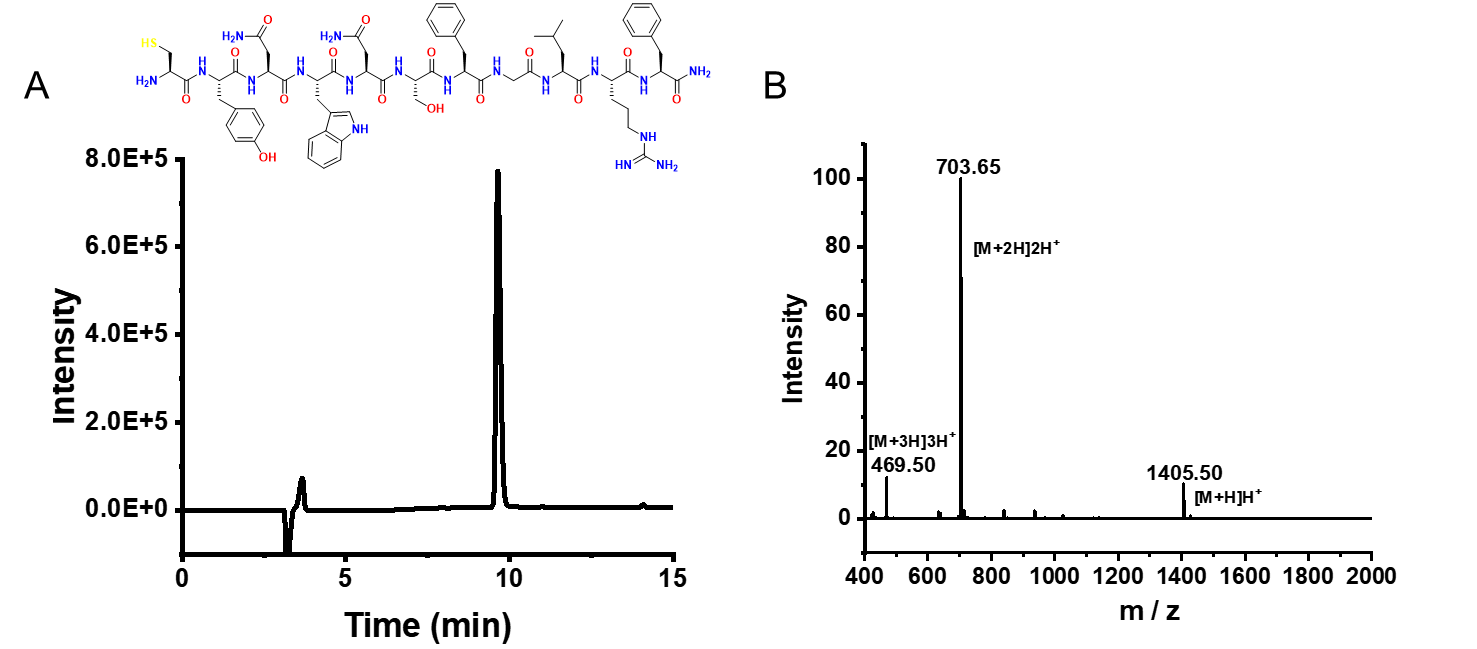


**Figure S5**. A) High performance liquid chromatography (HPLC) of Kisspeptin-10. B) Time of flight (TOF) mass spectra of Kisspeptin-10.

**
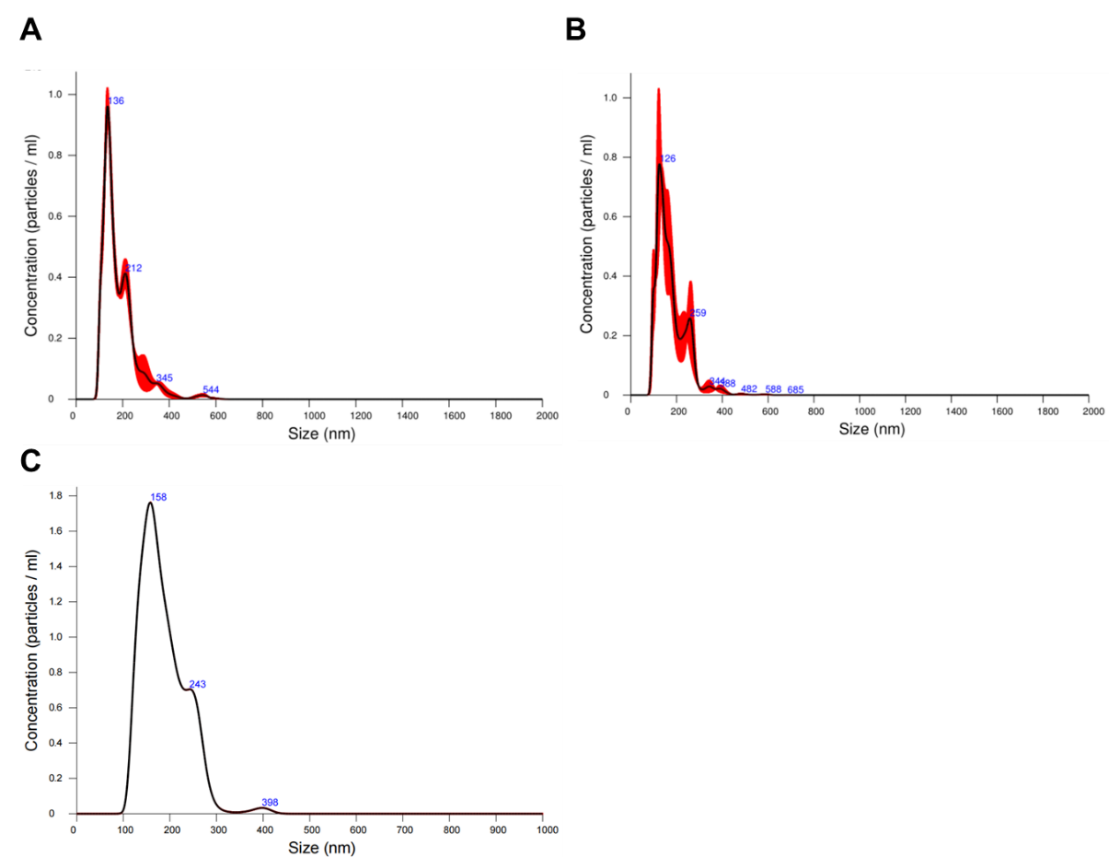
**

**Figure S6.** Size distribution of GEVs (A), KZCNs (B) and CZGE (C) from NTA.


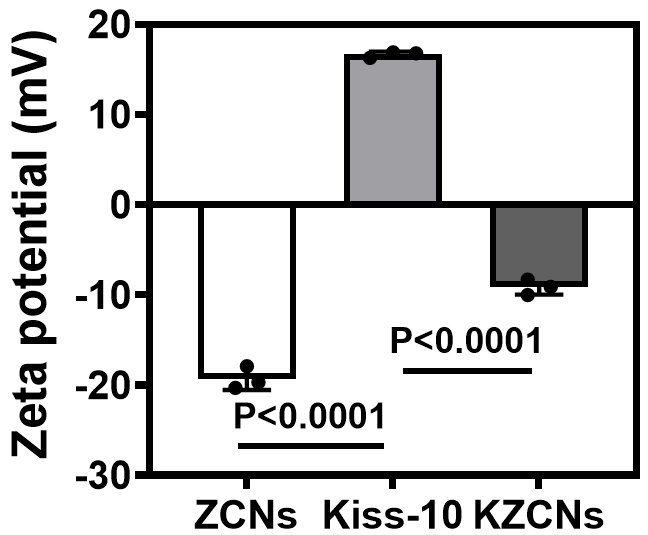


**Figure S7**. Zeta potential of different samples (*n* = 3). All statistical data are presented as mean **±** standard deviation. All statistical data are presented as mean ± standard deviation. Statistical analysis: one-way ANOVA followed by Tukey’s HSD post hoc test.


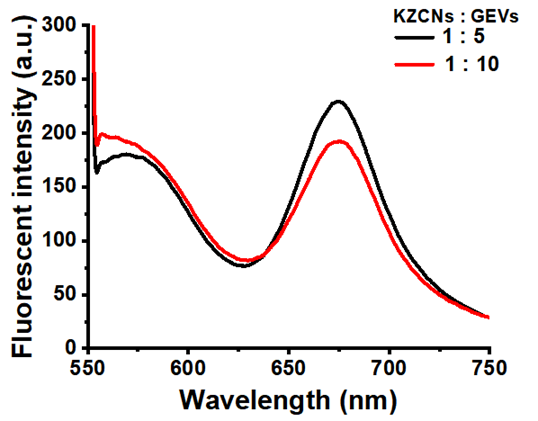


**Figure S8**. GEVs membrane labeled with fluorescent dyes (Dil and DiD) and fused with growing amounts of KZCNs. The fluorescence recovery of the donor (DiD) was measured to evaluate the fusion by FTIR spectra.


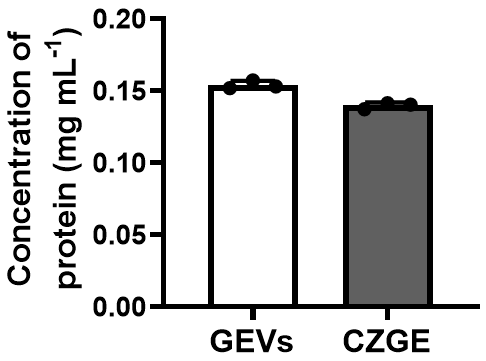


**Figure S9**. BCA quantification of GEVs and CZGE.


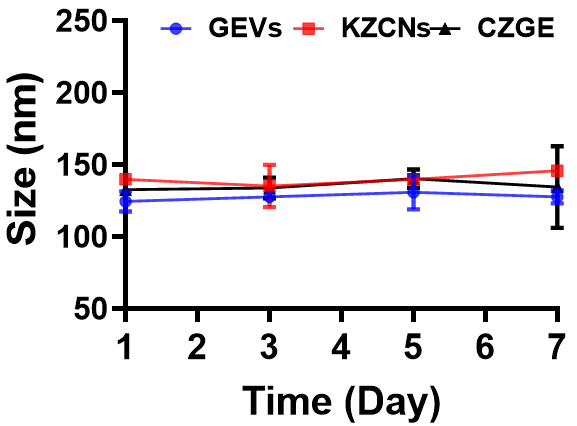


**Figure S10**. Stability of different samples over the period time in terms of size change at 37 ℃ (*n* = 3).


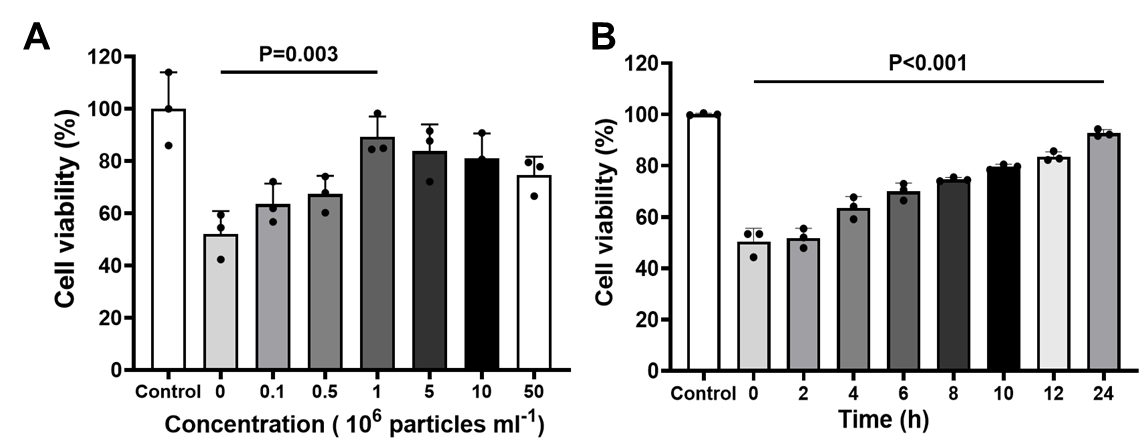


**Figure S11**. A) The cell viability of the stimulated GT1-7 cells treated by different concentrations of CZGE (*n* = 3). B) The cell viability of the stimulated GT1-7 cells treated by CZGE at different incubation times (*n* = 3). All statistical data are presented as mean ± standard deviation. Statistical analysis: one-way ANOVA followed by Tukey’s HSD post hoc test.


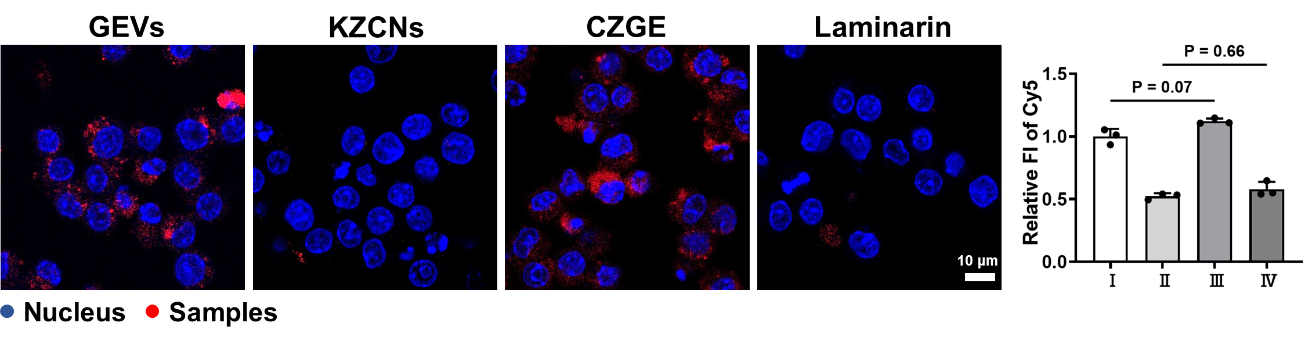


**Figure S12.** Representative fluorescent images and quantitative of different Cy5-labeled samples (red) in the stimulated RAW264.7 cells *in vitro* IEB model (I: GEVs, Ⅱ: KZCNs, Ⅲ: CZGE, Ⅳ: Laminarin, *n* = 3). All statistical data are presented as mean ± standard deviation. Statistical analysis: one-way ANOVA followed by Tukey’s HSD post hoc test.


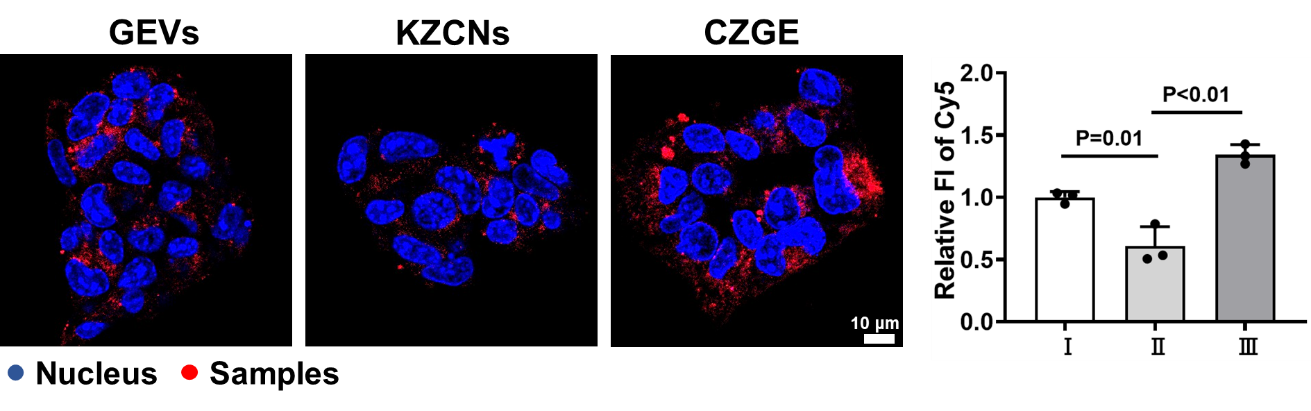


**Figure S13.** Representative fluorescent images and quantitative of different Cy5-labeled samples (red) in the stimulated GT1-7 cells *in vitro* BBB model (I: GEVs, Ⅱ: KZCNs, Ⅲ: CZGE, *n* = 3). All statistical data are presented as mean ± standard deviation. Statistical analysis: one-way ANOVA followed by Tukey’s HSD post hoc test.


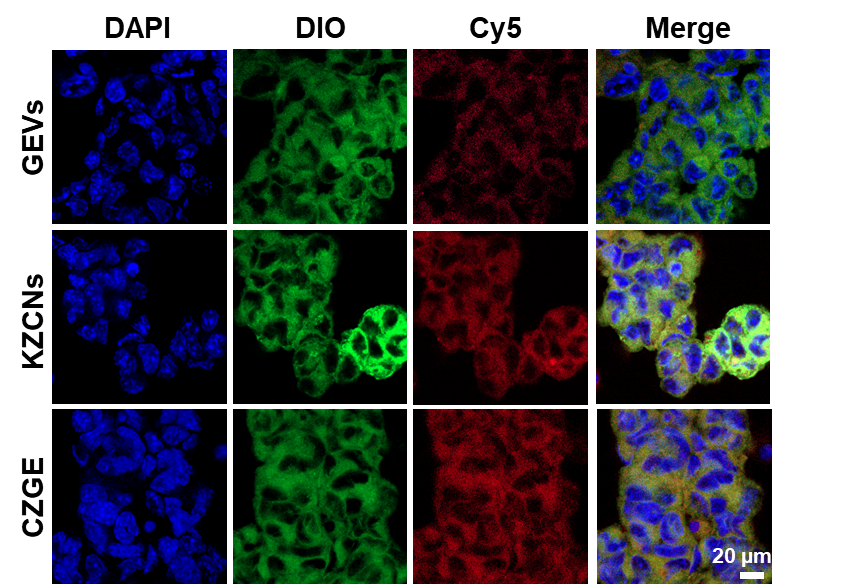


**Figure S14**. Representative CLSM images of the stimulated GT1-7 cells after treated by different samples (red: samples, blue: nucleus, green: cell membrane).


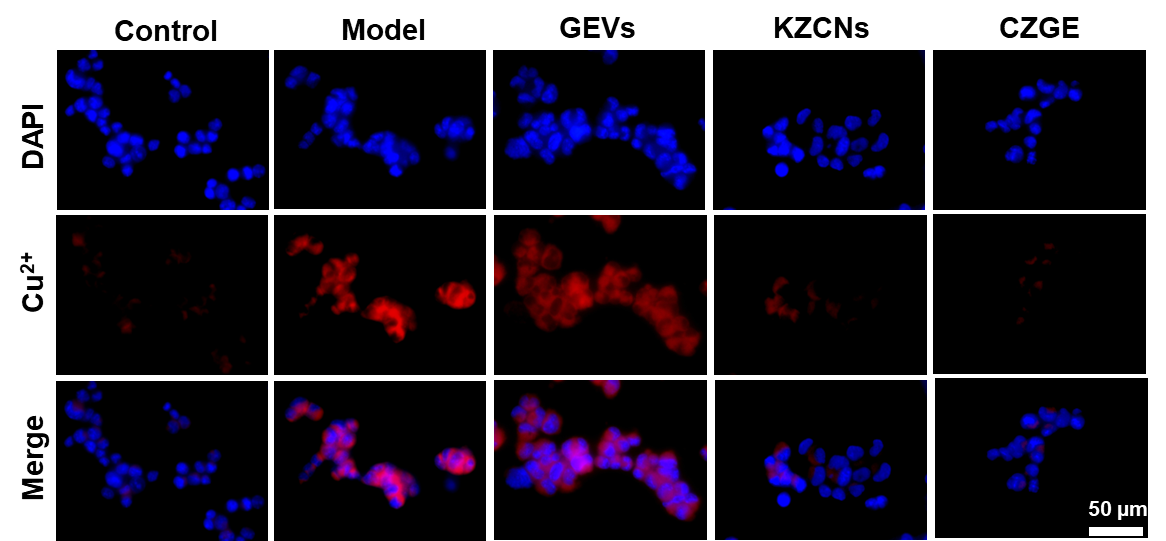


**Figure S15**. Representative fluorescent images of stimulated GT1-7 cells after uptake of different samples (blue: nucleus, green: cell membrane, red: Cu^2+^).

**
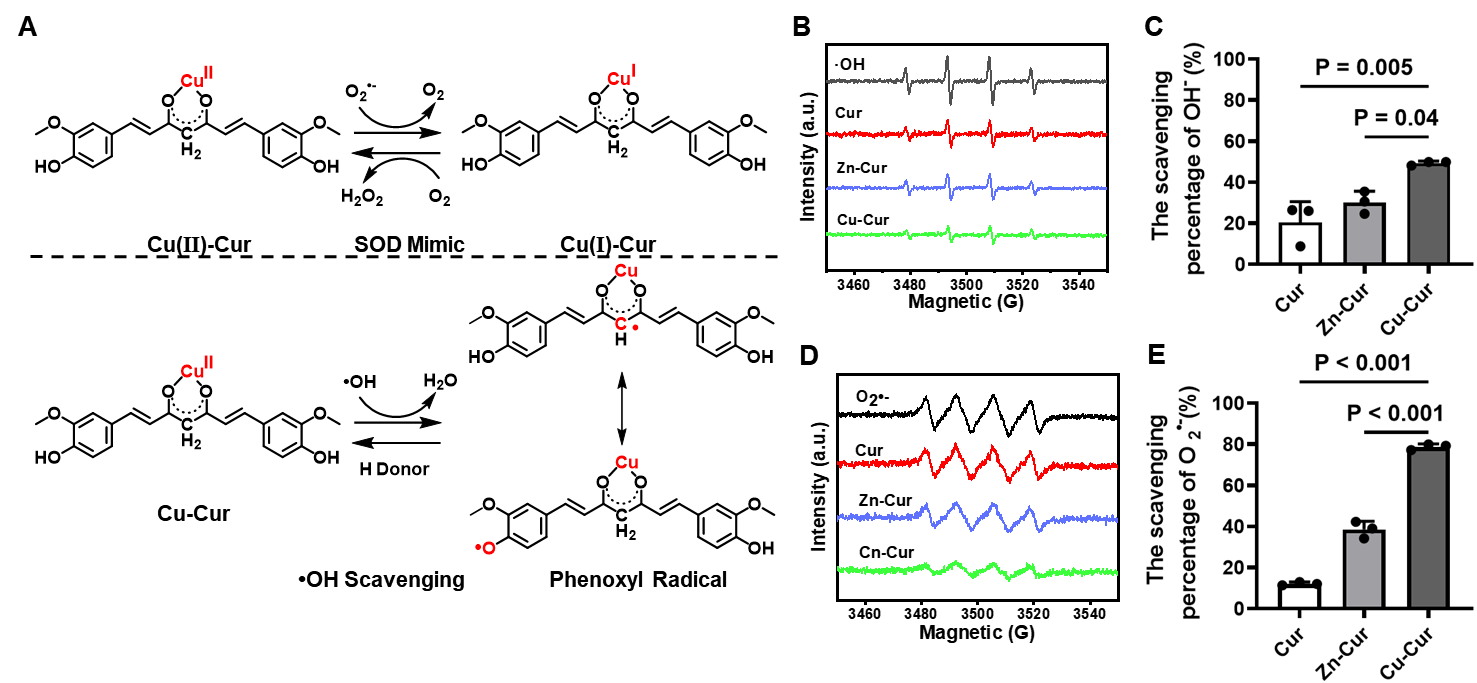
**

**Figure S16**. A) Schematics for the chemical mechanisms of O_2_^• −^and ^•^OH scavenging by Cu-Cur. B) ESR spectra of the O_2_^• −^ reaction system after different treatments. C) O_2_^• −^ inhibition rate of Cur, Zn-Cur, and Cu-Cur (*n* = 3). D) ESR spectra of Fenton reaction system after different treatments. E) ^•^OH inhibition rate of Cur, Zn-Cur, and Cu-Cur (*n* = 3). All statistical data are presented as mean ± standard deviation. Statistical analysis: one-way ANOVA followed by Tukey’s HSD post hoc test.


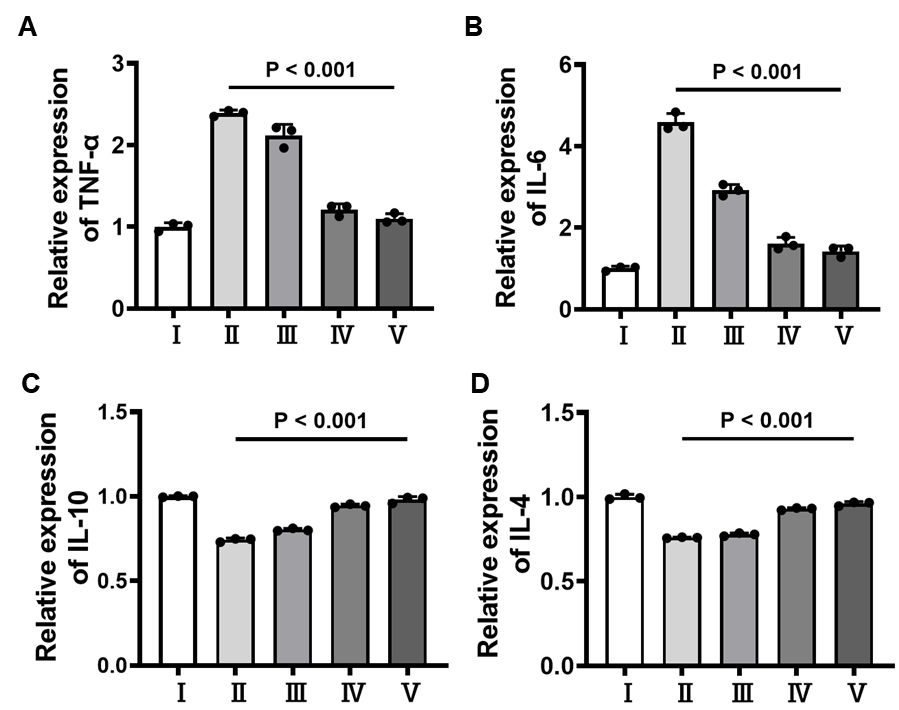


**Figure S17**. Relative quantification of TNF-α (A), IL-6 (B), IL-10 (C) and IL-4 (D) in GT1-7 cells by different treatments, in which the expression of control group was denoted as 1 (Ⅰ: Control, Ⅱ: Model, Ⅲ: GEVs, Ⅳ: KZCNs, Ⅴ: CZGE, *n* = 3). All statistical data are presented as mean ± standard deviation. Statistical analysis: one-way ANOVA followed by Tukey’s HSD post hoc test.


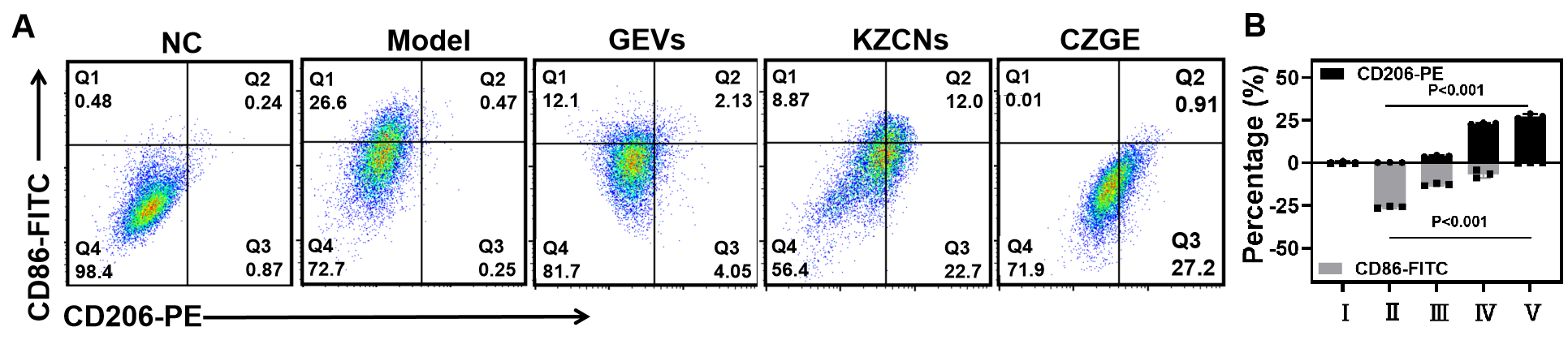


**Figure S18.** A) BV2 cells phenotype investigation treated by different samples via flow cytometry. B) The corresponding expression of CD86 and CD206 of BV2 cells under different condition (I: Control, Ⅱ: Model, Ⅲ: GEVs, Ⅳ: KZCNs, Ⅴ: CZGE, *n* = 3). All statistical data are presented as mean ± standard deviation. Statistical analysis: one-way ANOVA followed by Tukey’s HSD post hoc test.


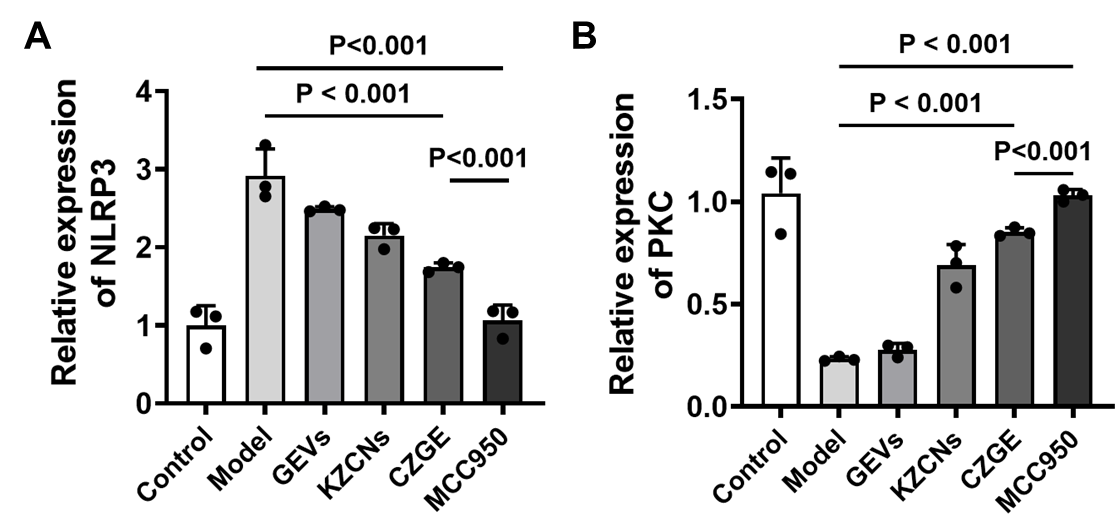


**Figure S19.** Relative quantification of NLRP3 (A) and PKC (B) in GT1-7 cells by different treatments, in which the expression of control group was denoted as 1 (*n* = 3). All statistical data are presented as mean ± standard deviation. Statistical analysis: one-way ANOVA followed by Tukey’s HSD post hoc test.


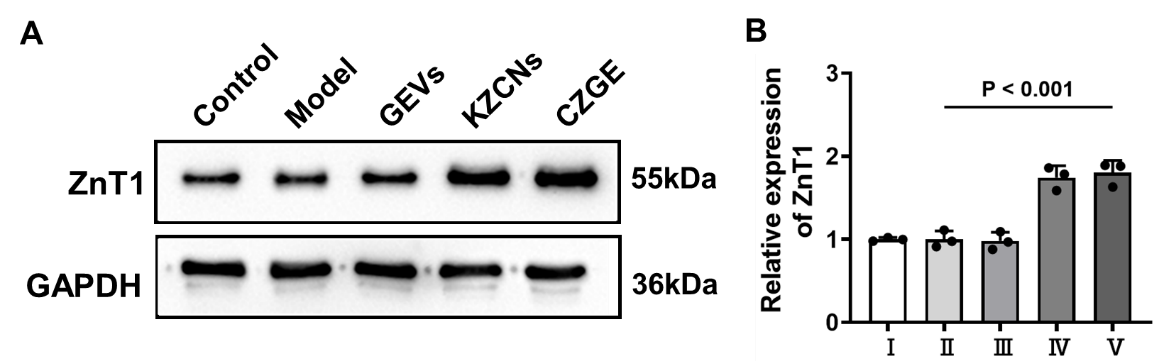


**Figure S20.** Representative western blot image (A) and quantification (B) of the relative protein expression of ZnT1, in which the expression of control group was denoted as 1 (I: Control, Ⅱ: Model, Ⅲ: GEVs, Ⅳ: KZCNs, Ⅴ: CZGE, *n* = 3). All statistical data are presented as mean ± standard deviation. Statistical analysis: one-way ANOVA followed by Tukey’s HSD post hoc test.

.


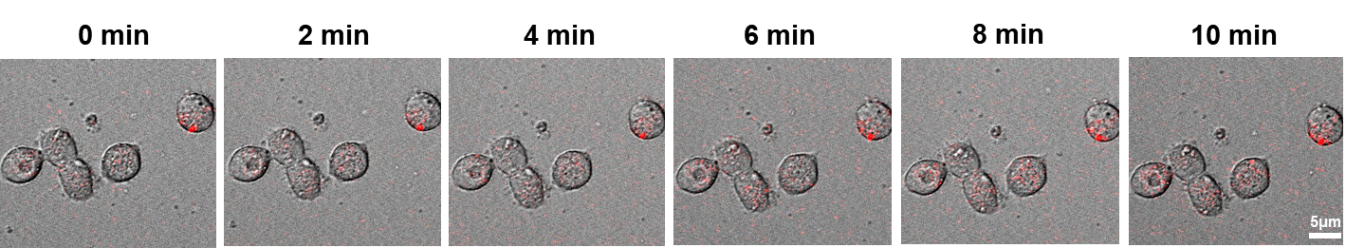


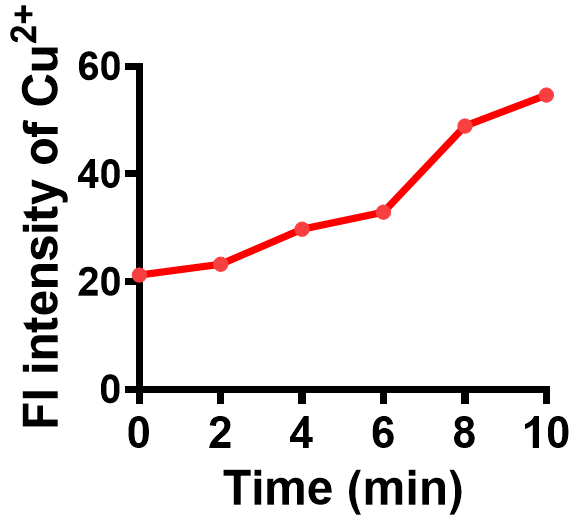


**Figure S21**. Time-course fluorescence microscopy images and quantification of GT1-7 cells after incubation with Cu^2+^ fluorescent probe (red) and addition of CuSO_4_.


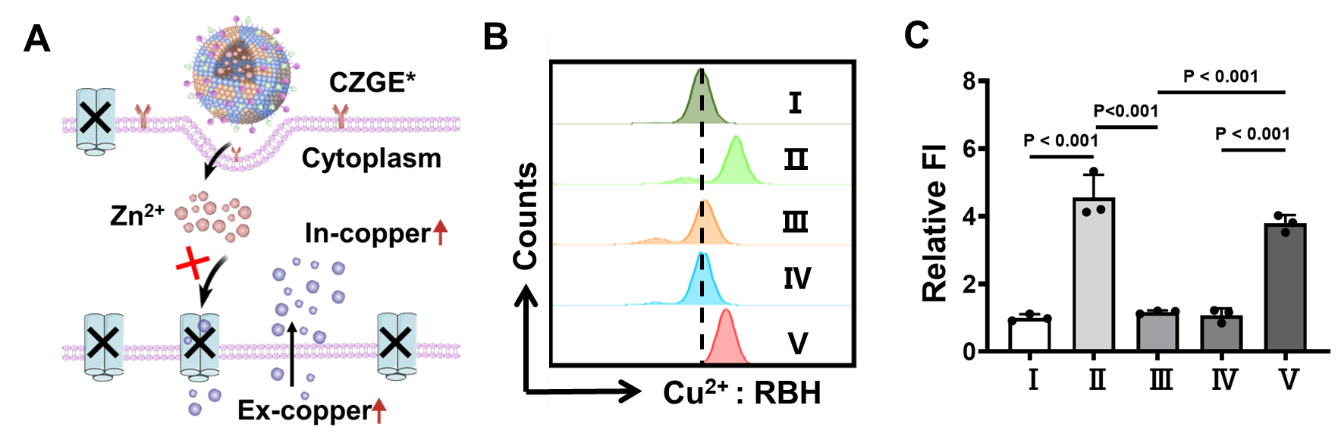


**Figure S22.** A) Schematic illustration of Zn^2+^ inhibiting ex-copper inflow via ZnT1. B, C) The intracellular Cu^2+^ levels investigation of GT1-7 cells under different conditions stained with Cu^2+^ fluorescence probe detected by flow cytometry (I: model group, Ⅱ: model group with another addition of CuSO_4_, Ⅲ: model group treated by CZGE* and another addition of CuSO_4_, Ⅳ: model group treated by si-ZnT1 and CZGE*, Ⅴ: model group treated by si-ZnT1 and CZGE*, and followed by another addition of CuSO_4_, *n* = 3). All statistical data are presented as mean ± standard deviation. Statistical analysis: one-way ANOVA followed by Tukey’s HSD post hoc test.

**
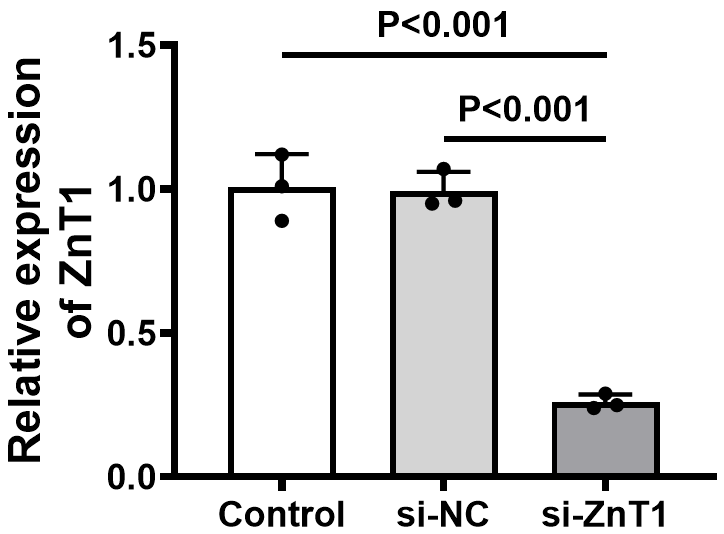
**

**Figure S23.** Transfection efficiency of ZnT1 (*n* = 3). All statistical data are presented as mean ± standard deviation. Statistical analysis: one-way ANOVA followed by Tukey’s HSD post hoc test.


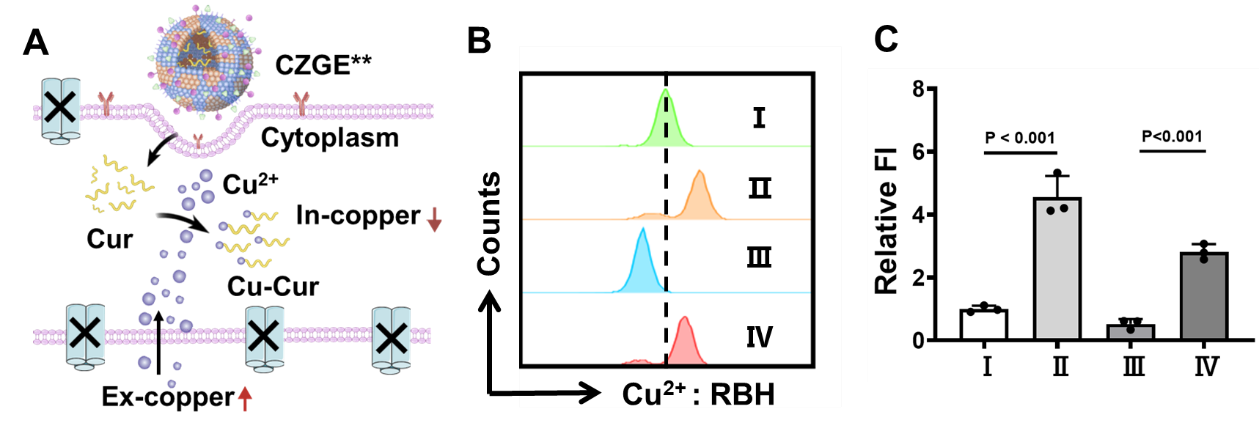


**Figure S24.** A) Schematic diagram illustrating that ZnT1 is the primary target for the action of Zn^2+^. B, C) The intracellular Cu^2+^ levels investigation of GT1-7 cells under different conditions stained with Cu^2+^ fluorescence probe detected by flow cytometry (I: model group, Ⅱ: model group with another addition of CuSO_4_, Ⅲ: model group treated by si-ZnT1 and CZGE**, Ⅳ: model group treated by si-ZnT1 and CZGE**, and followed by another addition of CuSO_4_, *n* = 3). All statistical data are presented as mean ± standard deviation. Statistical analysis: one-way ANOVA followed by Tukey’s HSD post hoc test.


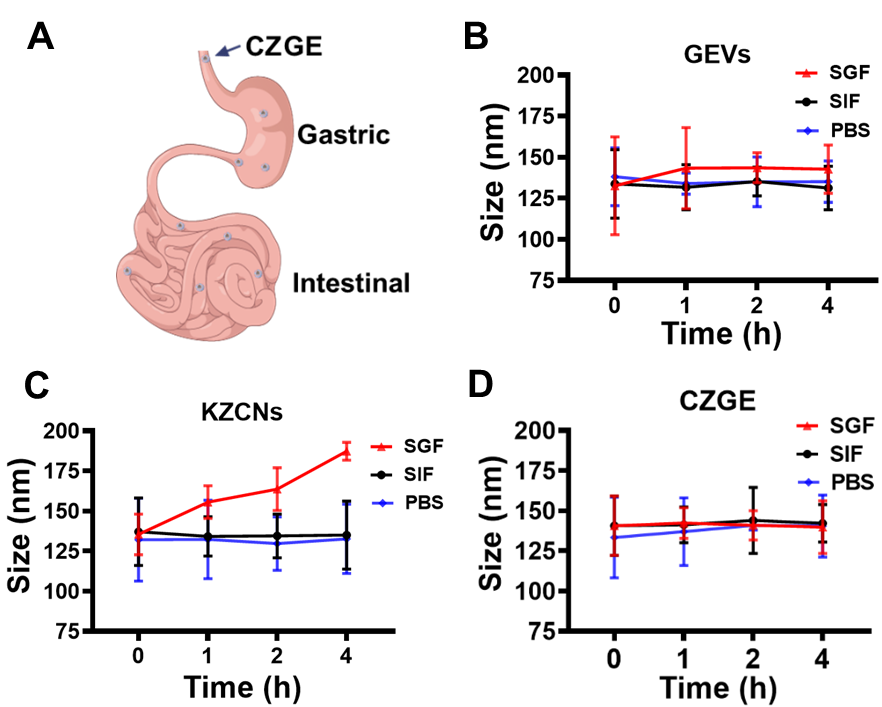


**Figure S25**. A) Schematic illustration of the gastrointestinal stability experiment. B-D)Sizes of GEVs (B), KZCNs (C) and CZGE (D) before and after incubation in PBS, SGF or SIF, measured using DLS (*n* = 3).


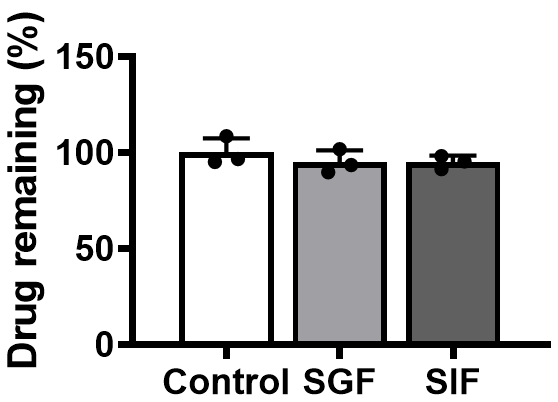


**Figure S26**. Zn-Cur remaining in CZGE before and after incubation in SGF or SIF (*n* = 3).


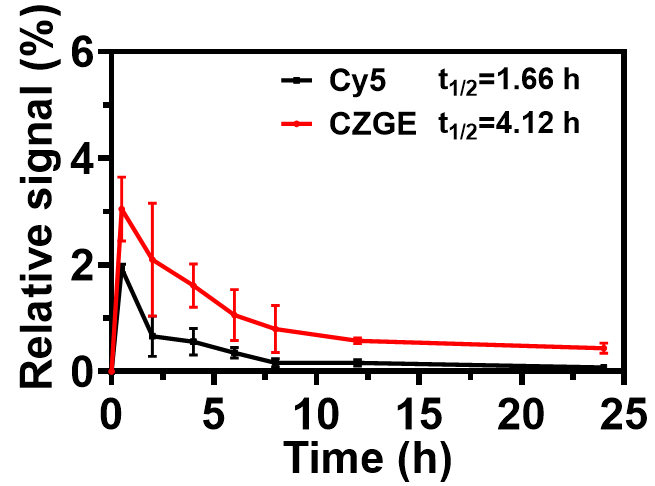


**Figure S27**. The corresponding signal intensities collected at different timepoints from the mice treated with different samples through oral gavage (*n* = 3).


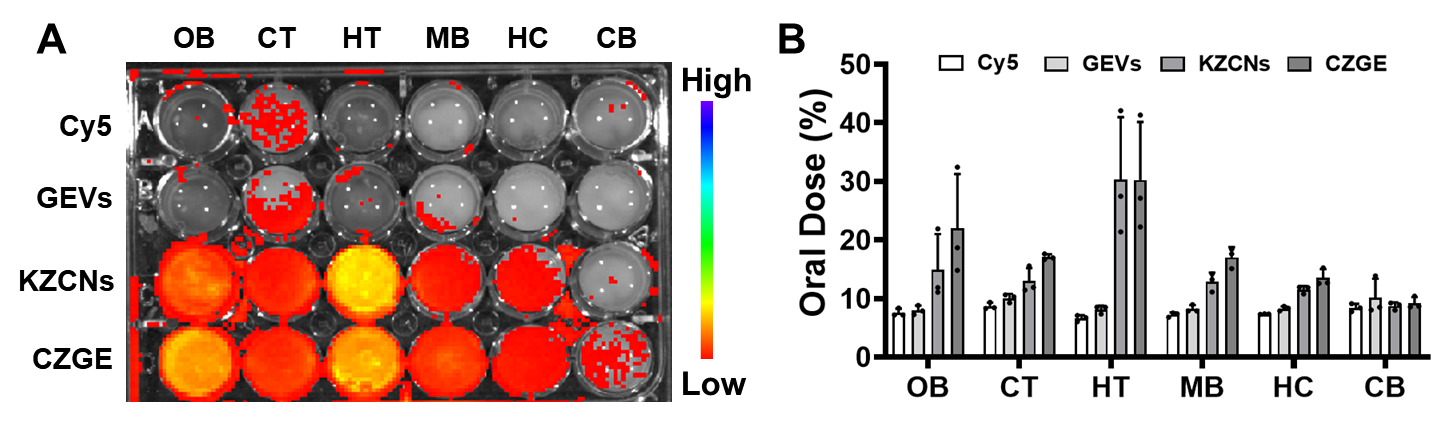


**Figure S28.** *Ex vivo* fluorescence imaging and quantitative analysis of olfactory bulb (OB), cortex (CT), hypothalamus (HT), midbrain (MB), hippocampus (HC), and cerebellum (CB) after 24 h (blank each brain region was used as a control, *n* = 3).


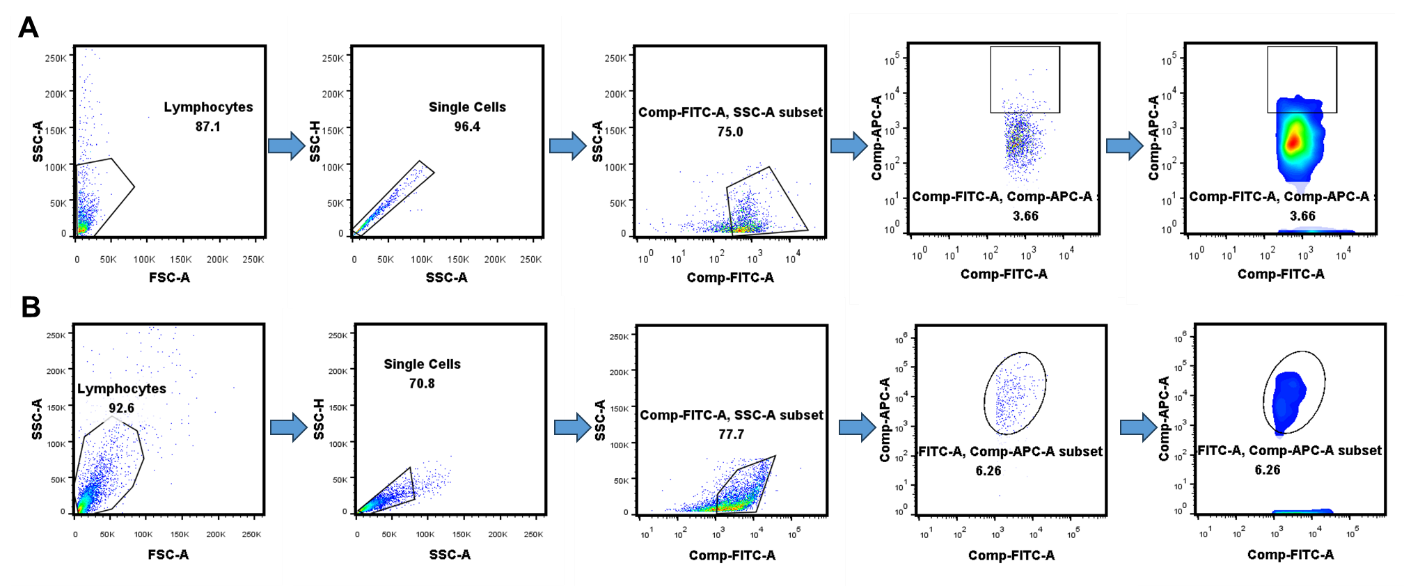


**Figure S29.** A) Gating strategy for Cy5^+^ Dectin-1^+^ cells analysis in ileum. B) Gating strategy for Cy5^+^ GnRH^+^ cells analysis in brain. The perfused hypothalamus tissues were digested and the dissociated cells were collected for flow cytometry analysis. 100,00 events were collected for each sample in the analysis. Live cells were chosen based on size in the FSC/SSC plots, and then single cells number was determined by SSCA/SSCH. On this basis, the proportion of cells co-expressing Cy5 and GnRH or Cy5 and Dectin-1 were selected by Cy5^+^ GnRH^+^ or Cy5^+^ Dectin-1^+^ gated sorting, which was presented on Figure 5H and 5L.


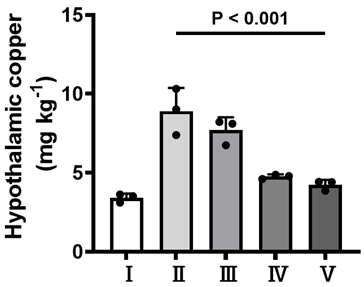


**Figure S30**. The content of copper in hypothalamic tissue of mice was detected by inductively coupled plasma mass spectrometry (ICP-MS). (Ⅰ: Control, Ⅱ: Model, Ⅲ: GEVs, Ⅳ: KZCNs, Ⅴ: CZGE, *n* = 3). All statistical data are presented as mean ± standard deviation. Statistical analysis: one-way ANOVA followed by Tukey’s HSD post hoc test.


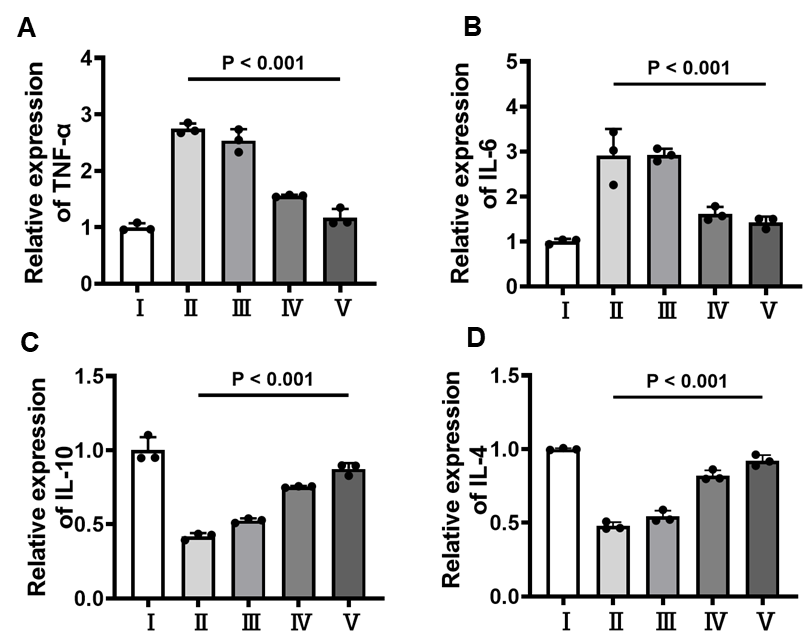


**Figure S31**. Relative quantification of TNF-α (A), IL-6 (B), IL-10 (C) and IL-4 (D) in hypothalamic tissue of mice by different treatments, in which the expression of control group was denoted as 1 (Ⅰ: Control, Ⅱ: Model, Ⅲ: GEVs, Ⅳ: KZCNs, Ⅴ: CZGE, *n* = 3). All statistical data are presented as mean ± standard deviation. Statistical analysis: one-way ANOVA followed by Tukey’s HSD post hoc test.


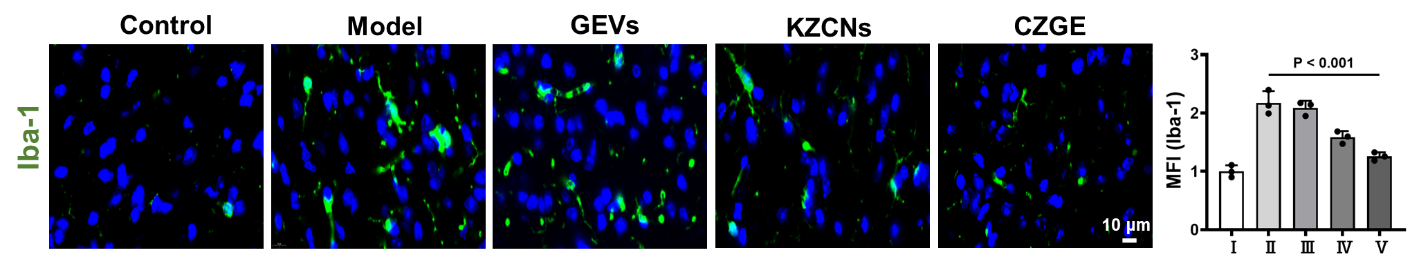


**Figure S32**. Immunofluorescence staining and quantitative analysis of Iba-1 (green) in the hypothalamus of mice by different treatment, in which the expression of control group was denoted as 1 (I: Control, Ⅱ: Model, Ⅲ: GEVs, Ⅳ: KZCNs, Ⅴ: CZGE, *n* = 3). All statistical data are presented as mean ± standard deviation. Statistical analysis: one-way ANOVA followed by Tukey’s HSD post hoc test.


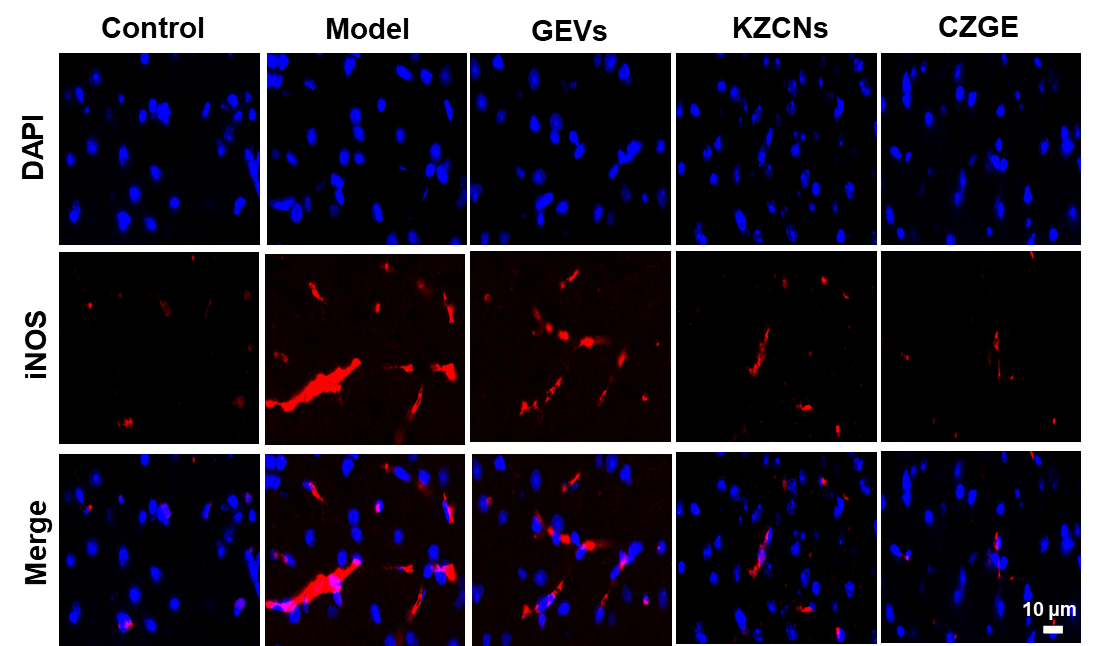


**Figure S33.** Representative immunostaining images of iNOS (red) in hypothalamic region of mice after administration of different samples.


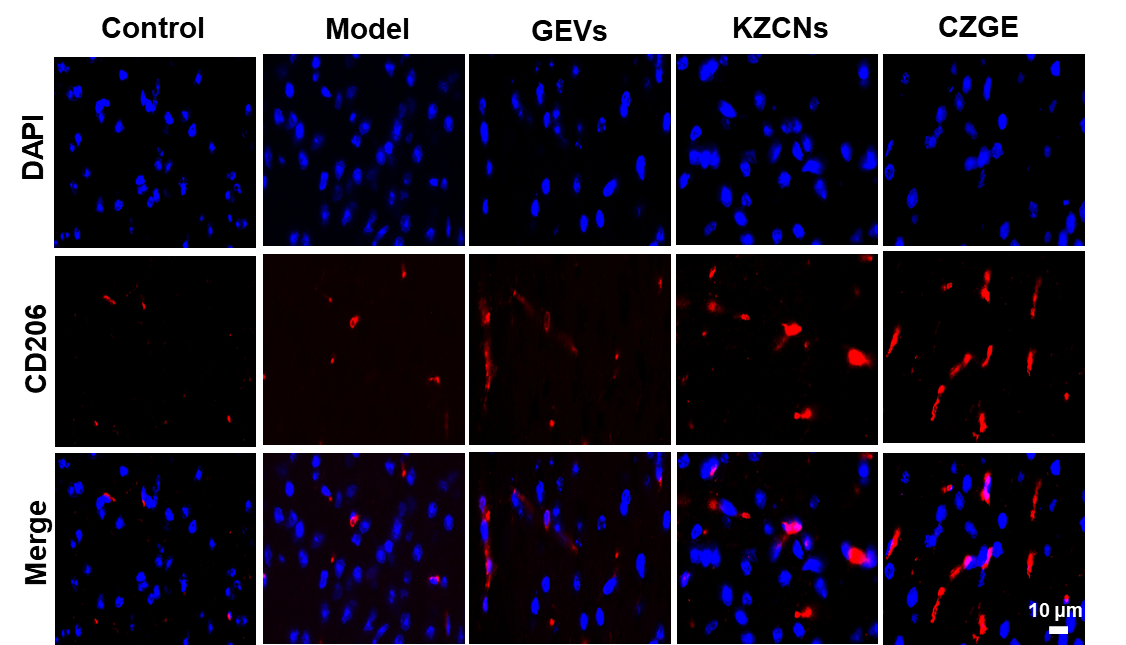


**Figure S34.** Representative immunostaining images of CD206 (red) in hypothalamic region of mice after administration of different samples.


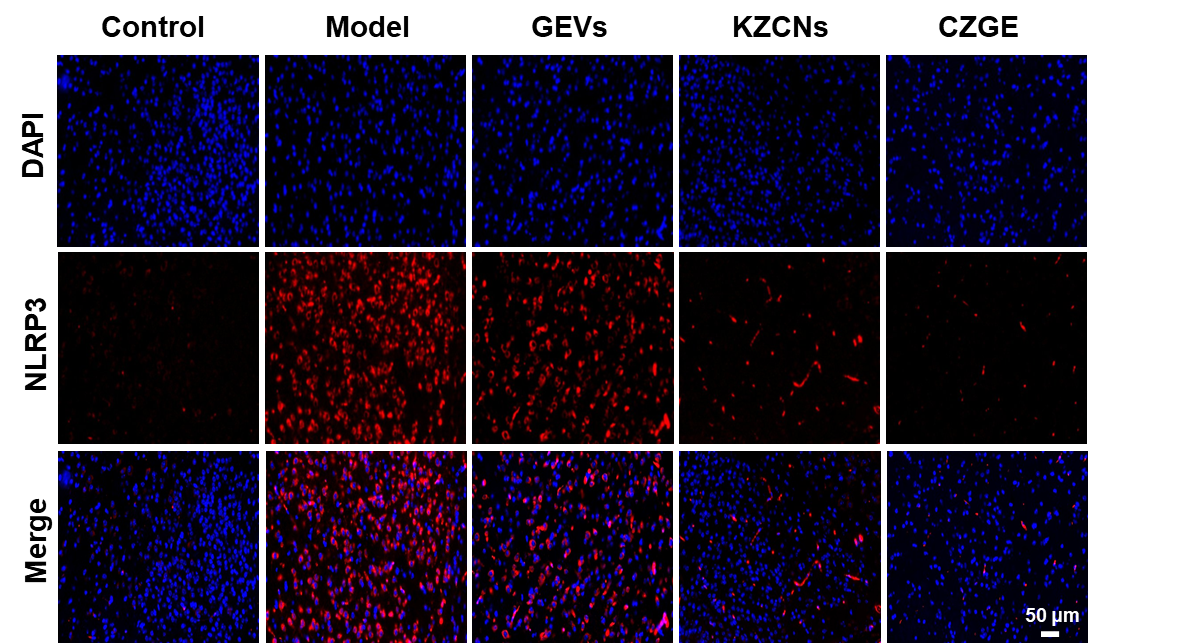


**Figure S35.** Representative immunostaining images of NLRP3 (red) in hypothalamic region of mice after administration of different samples.

**
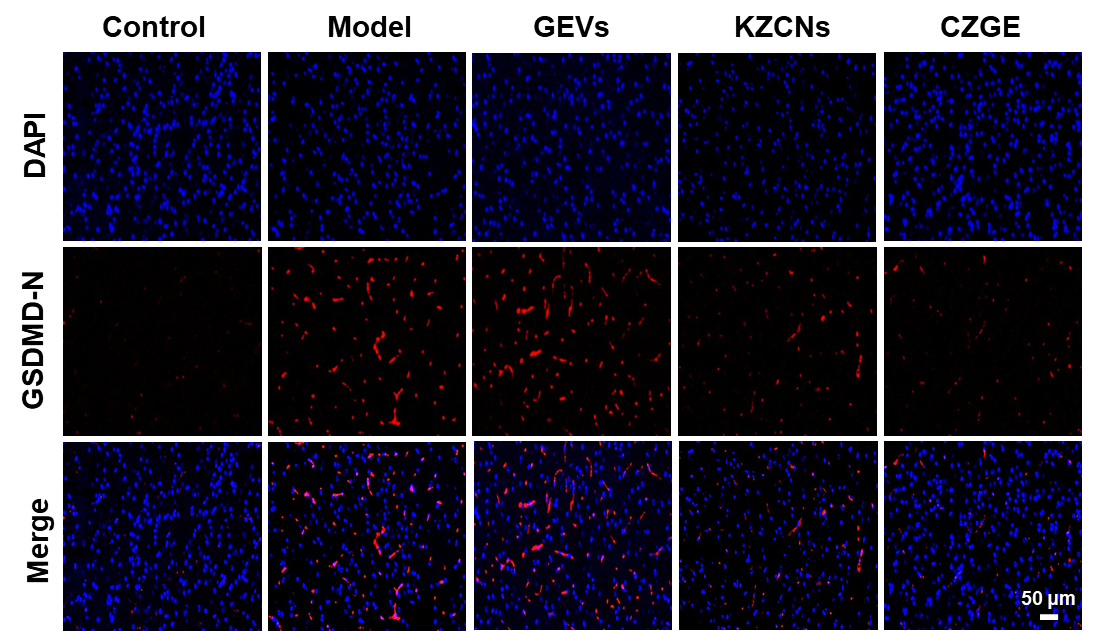
**

**Figure S36.** Representative immunostaining images of GSDMD-N (red) in hypothalamic region of mice after administration of different samples.


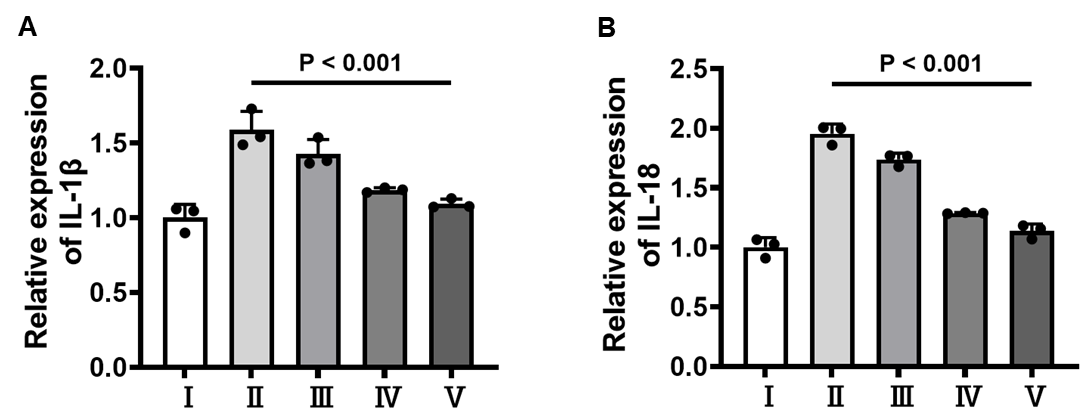


**Figure S37**. Relative quantification of IL-1*β* (A) and IL-18 (B) in hypothalamic tissue of mice by different treatments, in which the expression of control group was denoted as 1 (Ⅰ: Control, Ⅱ: Model, Ⅲ: GEVs, Ⅳ: KZCNs, Ⅴ: CZGE, *n* = 3). All statistical data are presented as mean ± standard deviation. Statistical analysis: one-way ANOVA followed by Tukey’s HSD post hoc test.


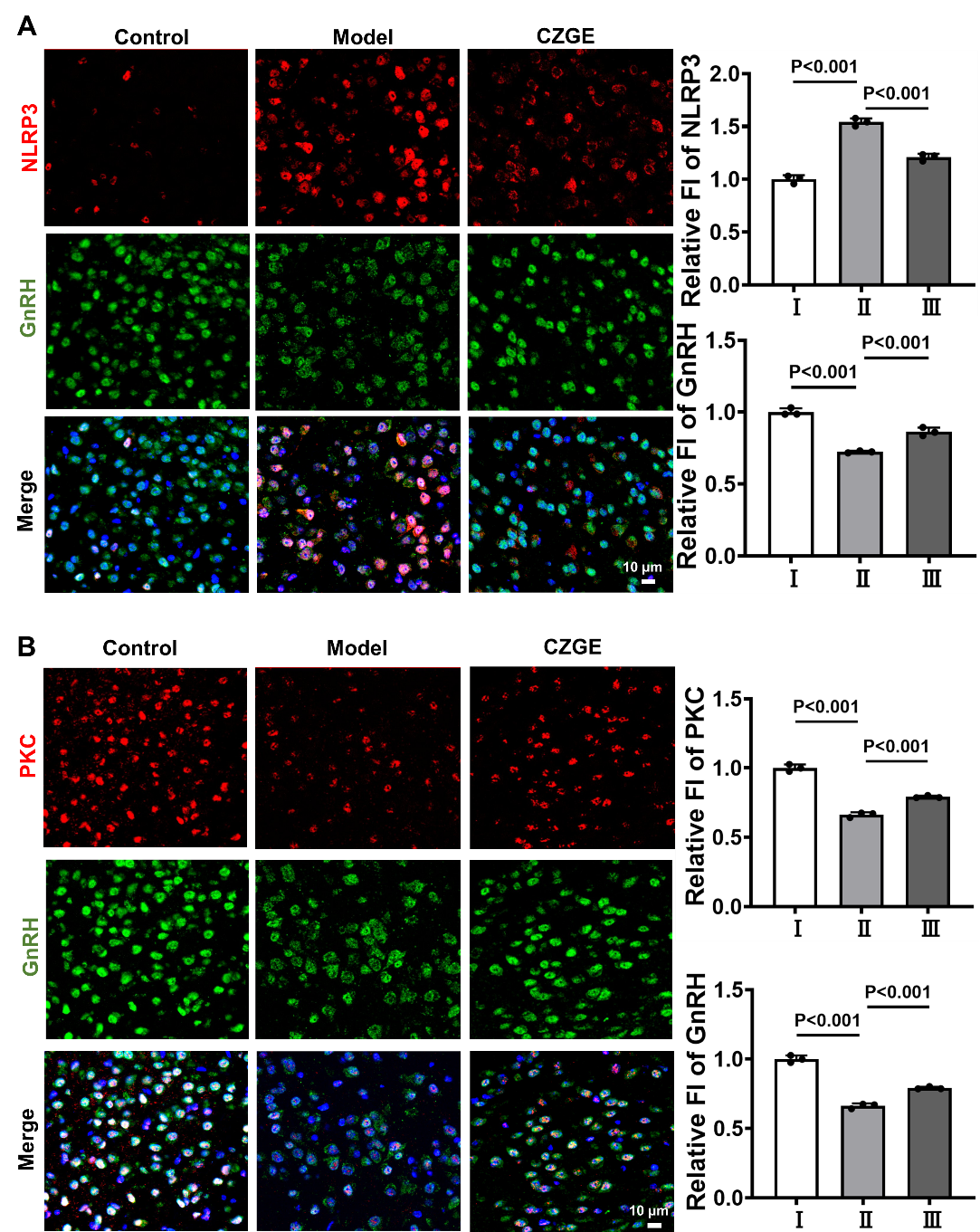


**Figure S38.** A) Immunofluorescence staining and quantitative analysis of NLRP3 (red) and GnRH (green) in the hypothalamus of mice by different treatment, in which the expression of control group was denoted as 1. B) Immunofluorescence staining and quantitative analysis of PKC (red) and GnRH (green) in the hypothalamus of mice by different treatment, in which the expression of control group was denoted as 1. (Ⅰ: Control, Ⅱ: Model, Ⅲ: CZGE, *n* = 3). All statistical data are presented as mean ± standard deviation. Statistical analysis: one-way ANOVA followed by Tukey’s HSD post hoc test.


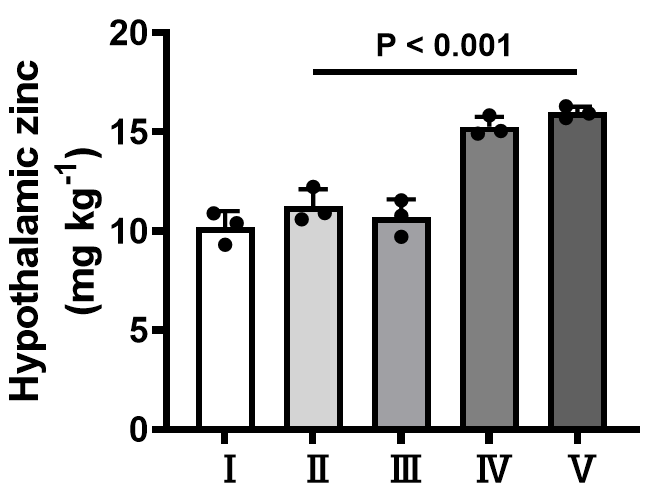


**Figure S39**. The content of zinc in hypothalamic tissue of mice was detected by ICP-MS (Ⅰ: Control, Ⅱ: Model, Ⅲ: GEVs, Ⅳ: KZCNs, Ⅴ: CZGE, *n* = 3). All statistical data are presented as mean ± standard deviation. Statistical analysis: one-way ANOVA followed by Tukey’s HSD post hoc test.


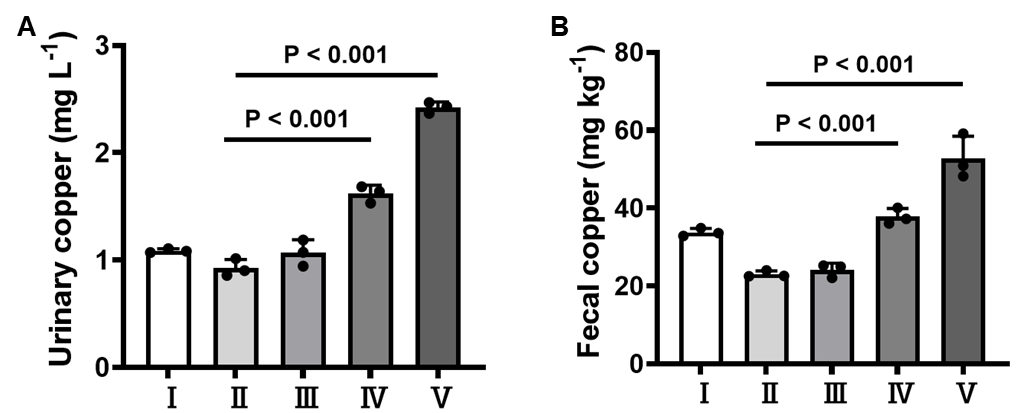


**Figure S40**. The content of copper in urinary (A) and fecal (B) of mice was detected by ICP-MS (Ⅰ: Control, Ⅱ: Model, Ⅲ: GEVs, Ⅳ: KZCNs, Ⅴ: CZGE, *n* = 3). All statistical data are presented as mean ± standard deviation. Statistical analysis: one-way ANOVA.


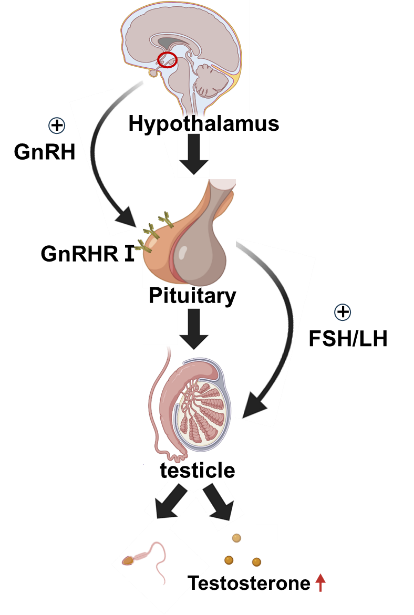


**Figure S41.** Schematic diagram illustrating the regulation of reproduction by GnRH through the HPT axis.


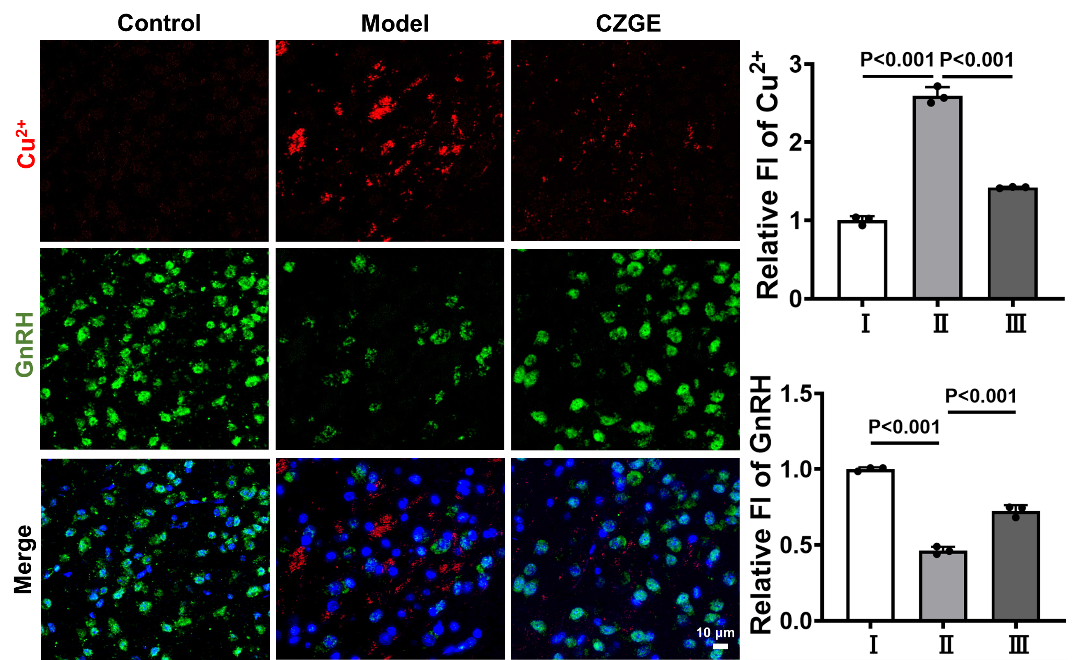


**Figure S42.** Immunofluorescence staining and quantitative analysis of Cu^2+^ (red) and GnRH (green) in the hypothalamus of mice by different treatment, in which the expression of control group was denoted as 1 (Ⅰ: Control, Ⅱ: Model, Ⅲ: CZGE, *n* = 3). All statistical data are presented as mean ± standard deviation. Statistical analysis: one-way ANOVA followed by Tukey’s HSD post hoc test.


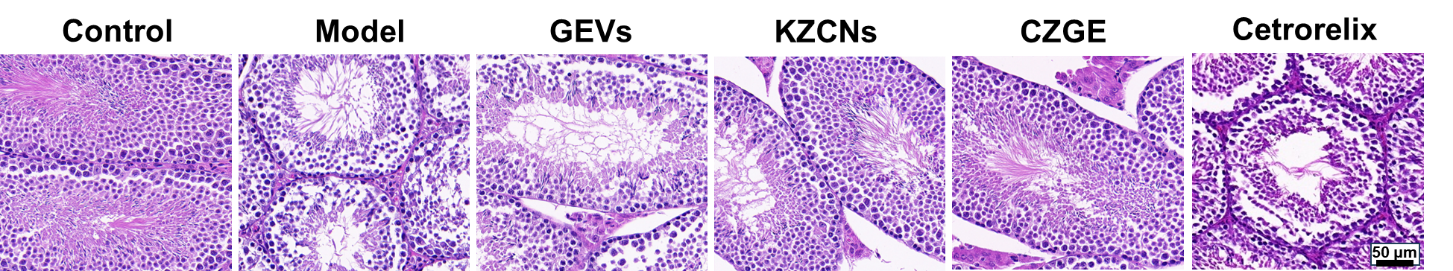


**Figure S43.** Representative H&E staining images of testicle from the different groups of mice.


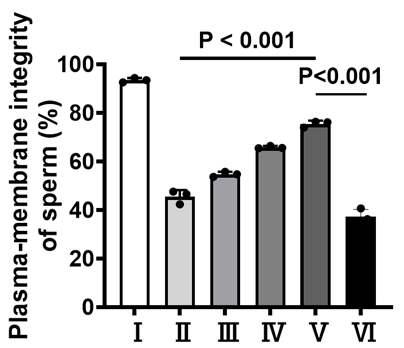


**Figure S44.** The sperm plasma-membrane integrity for the mice after different treatments by flow cytometry (I: Control, Ⅱ: Model, Ⅲ: GEVs, Ⅳ: KZCNs, Ⅴ: CZGE, Ⅵ: cetrorelix, *n* = 3). All statistical data are presented as mean ± standard deviation. Statistical analysis: one-way ANOVA followed by Tukey’s HSD post hoc test.


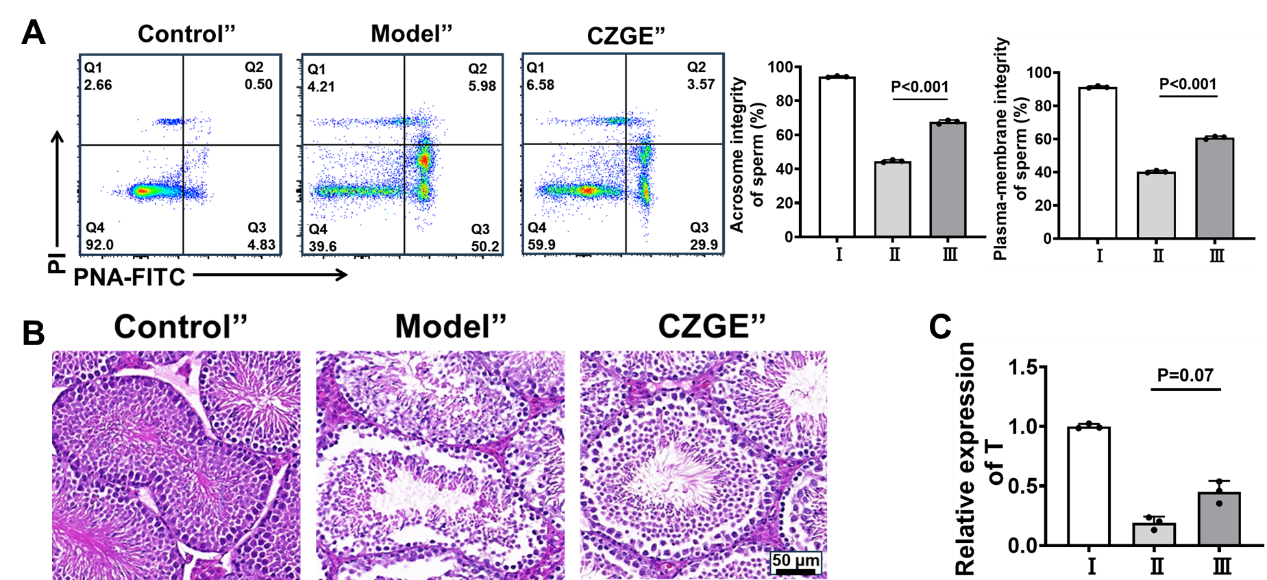


**Figure S45.** A) The sperm plasma-membrane and acrosome integrity for the mice of long-term effects after different treatments by flow cytometry. B) Representative H&E staining images of testicle from the different groups for the mice of long-term effects. C) Relative quantification of T in the serum of mice by different treatments of long-term effects, in which the expression of control group was denoted as 1 (I: Control’’, Ⅱ: Model’’, Ⅲ: CZGE’’, *n* = 3). All statistical data are presented as mean ± standard deviation. Statistical analysis: one-way ANOVA followed by Tukey’s HSD post hoc test.


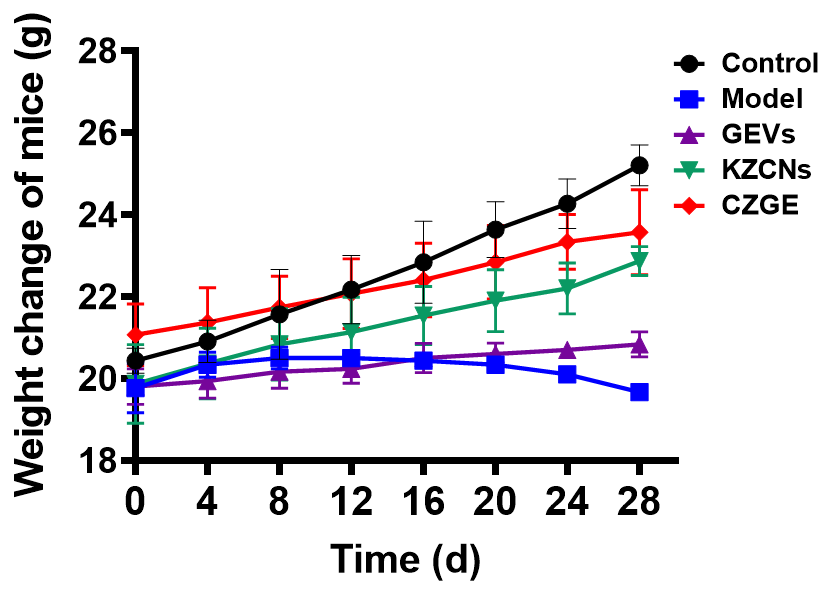


**Figure S46**. The body weight of mice treated by different samples treatment (*n* = 3). All statistical data are presented as mean ± standard deviation. Statistical analysis: two-way ANOVA.


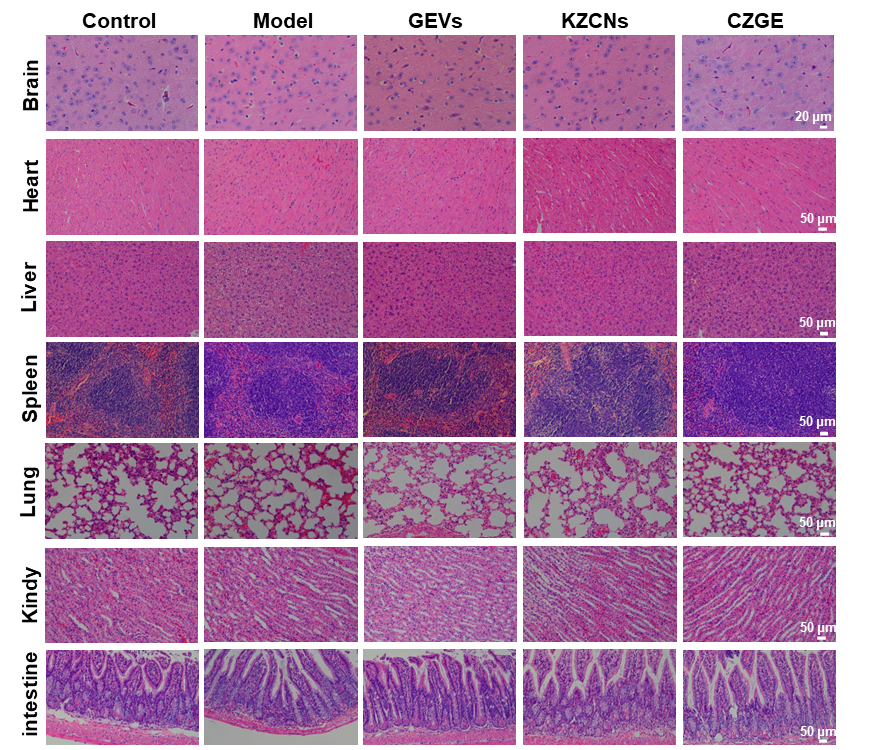


**Figure S47.** Representative H&E staining images of brain and major organs**.** Including the heart, liver, spleen, lung, kidney and intestine from the different groups of mice.


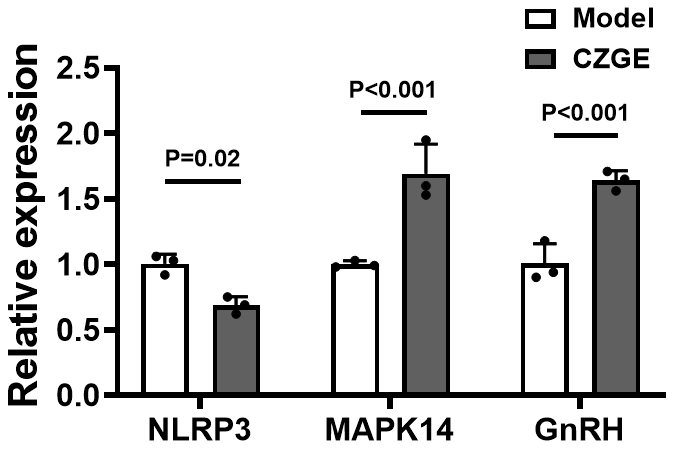


**Figure S48.** Relative mRNA expression of NLRP3, MAPK14 and GnRH in the hypothalamus from treated with different samples, in which the content in the model group was denoted as 1 (*n* = 3). All statistical data were presented as mean ± standard deviation. Statistical analysis: one-way ANOVA.

**Table S1.** Blood routine analysis of mice after being treated with different samples.

| Group | Control | Model | GEVs | KZCNs | CZGE |
| --- | --- | --- | --- | --- | --- |
| White blood cell count  (10^9^ L^-1^) | 1.4 | 0.7 | 1.2 | 0.7 | 0.7 |
| Lymphocyte count (10^9^ L^-1^) | 0.2 | 0.6 | 1 | 0.6 | 0.6 |
| Neutrophil count (10^9^ L^-1^) | 1 | 0.1 | 0.2 | 0.1 | 0.1 |
| Lymphocyte percentage (%) | 13 | 16.3 | 12.5 | 10.4 | 15.3 |
| Neutrophil percentage (%) | 72.2 | 81.6 | 85.4 | 88.3 | 83 |
| Red blood cell count  (1012 L^-1^) | 6.09 | 7.11 | 7.47 | 7.80 | 9.13 |
| Hemoglobin ( g L^-1^) | 105 | 97 | 10.1 | 105 | 123 |
| Mean erythrocyte hemoglobin concentration  (g L^-1^) | 262 | 277 | 244 | 206 | 272 |
| Platelet count (10^9^ L^-1^) | 91 | 98 | 82 | 85 | 100 |
| Platelet accumulation (%) | 0.046 | 0.076 | 0.045 | 0.044 | 0.054 |

**Table S2.** The sequences of qPCR primer (5'→3')

| Gene | Forward primer (5'→3') | Reverse primer (5'→3') |
| --- | --- | --- |
| *β*-actin | AGTGTGACGTTGACATCCGT | TGCTAGGAGCCAGAGCAGTA |
| NLRP3 | TCAACCTGCTGATTGAACCT | ACAAATTTCTCTGAGGCACTG |
| MAPK14 | AGCTGTTGACCGGAAGAACG | ATGTAGTTTCTTGCCTCATGGC |
| GnRH | TCACATCCAAACAGATGGGCA | GGGCCAGTGCATCTACATCTT |
| ZnT1 | ACCAGGCAGAGCCAGAA | GCAGAAACACTCCTCGCATA |
